# The tumor suppressor BRCA1-BARD1 complex localizes to the synaptonemal complex and regulates recombination under meiotic dysfunction in *Caenorhabditis elegans*

**DOI:** 10.1371/journal.pgen.1007701

**Published:** 2018-11-01

**Authors:** Qianyan Li, Takamune T. Saito, Marina Martinez-Garcia, Alison J. Deshong, Saravanapriah Nadarajan, Katherine S. Lawrence, Paula M. Checchi, Monica P. Colaiacovo, JoAnne Engebrecht

**Affiliations:** 1 Department of Molecular and Cellular Biology, University of California Davis; Davis CA, United States of America; 2 Department of Genetics, Harvard Medical School; Boston, MA, United States of America; The University of North Carolina at Chapel Hill, UNITED STATES

## Abstract

Breast cancer susceptibility gene 1 (BRCA1) and binding partner BRCA1-associated RING domain protein 1 (BARD1) form an essential E3 ubiquitin ligase important for DNA damage repair and homologous recombination. The *Caenorhabditis elegans* orthologs, BRC-1 and BRD-1, also function in DNA damage repair, homologous recombination, as well as in meiosis. Using functional GFP fusions we show that in mitotically-dividing germ cells BRC-1 and BRD-1 are nucleoplasmic with enrichment at foci that partially overlap with the recombinase RAD-51. Co-localization with RAD-51 is enhanced under replication stress. As cells enter meiosis, BRC-1-BRD-1 remains nucleoplasmic and in foci, and beginning in mid-pachytene the complex co-localizes with the synaptonemal complex. Following establishment of the single asymmetrically positioned crossover on each chromosome pair, BRC-1-BRD-1 concentrates to the short arm of the bivalent. Localization dependencies reveal that BRC-1 and BRD-1 are interdependent and the complex fails to properly localize in both meiotic recombination and chromosome synapsis mutants. Consistent with a role for BRC-1-BRD-1 in meiotic recombination in the context of the synaptonemal complex, inactivation of BRC-1 or BRD-1 enhances the embryonic lethality of mutants defective in chromosome synapsis. Our data suggest that under meiotic dysfunction, BRC-1-BRD-1 stabilizes the RAD-51 filament and alters the recombination landscape; these two functions can be genetically separated from BRC-1-BRD-1’s role in the DNA damage response. Together, we propose that BRC-1-BRD-1 serves a checkpoint function at the synaptonemal complex where it monitors and modulates meiotic recombination.

## Author summary

Our genomes are passed down from one generation to the next through the specialized cell division program of meiosis. Meiosis is highly regulated to coordinate both the large scale chromosomal and fine scale DNA events to ensure fidelity. While the tumor suppressor BRCA1-BARD1 is essential for genome integrity, its specific role in meiosis has been difficult to uncover. Taking advantage of attributes of the *Caenorhabditis elegans* system, we have analyzed the function of the BRCA1-BARD1 complex in meiosis in this simple metazoan. We find that BRCA1 and BARD1 localize dynamically to the proteinaceous structure that aligns maternal and paternal chromosomes, where it regulates homologous recombination. Although BRCA1 and BARD1 mutants have only subtle meiotic defects, we show that this complex plays critical roles in meiotic recombination when meiosis is perturbed, and this is separable from BRCA1-BARD1’s function in response to DNA damage in somatic cells. These results highlight the complexity of ensuring accurate transmission of the genome and uncover the requirement for this conserved complex in meiosis.

## Introduction

BRCA1 was identified twenty-eight years ago as the causative agent of early-onset familial breast cancer [1]. Subsequently, BRCA1 was shown to interact with BARD1 through their RING domains [2], to form an E3 ubiquitin ligase, which adds the small polypeptide ubiquitin to protein substrates [3] (hereafter referred to as BRCA1-BARD1). While BRCA1-BARD1 has been extensively studied with respect to its crucial tumor suppressor activities, we still do not fully understand how this protein complex mediates the diverse functions that have been ascribed to it (*e*.*g*., DNA metabolism, checkpoint signaling, chromatin dynamics, centrosome amplification, and transcriptional and translational regulation [4, 5]). This is due in part to the diversity of protein-protein interactions involved in generating numerous distinct BRCA1-BARD1 multi-protein complexes [6]. An additional impediment to understanding BRCA1-BARD1 function is that the corresponding mouse knockouts are embryonic lethal [7, 8].

The simple metazoan *Caenorhabditis elegans* offers several advantages to the study of this key complex. First, unlike in mammals, *C*. *elegans* BRCA1 and BARD1 orthologs, BRC-1 and BRD-1, are not essential yet play critical roles in DNA replication and the DNA damage response, as well as in homologous recombination, which is critical for repairing programmed double strand breaks (DSBs) during meiosis [9–14]. Additionally, attributes of the *C*. *elegans* system, including sophisticated genetics, ease of genome editing, and the spatio-temporal organization of the germ line allow us to overcome some challenges inherent in studying this complex in mammalian meiosis.

Meiosis is essential for sexual reproduction and results in the precise halving of the genome for packaging into gametes. During meiosis, homologous chromosomes are connected by crossover recombination to facilitate their alignment and segregation on the meiotic spindle. Recombination is integrated and reinforced with chromosome pairing and synapsis, although the extent of dependencies of these critical meiotic processes are distinct in different organisms (reviewed in [15, 16]). While it is well established that BRCA1-BARD1 plays an important role in DNA repair and recombination [5], the specific function of BRCA1-BARD1 in meiotic recombination is not known. In mice, partial deletions of BRCA1 result in early apoptosis of male germ cells due to failures in meiotic sex chromosome inactivation [17, 18]. BRCA1 has been shown to co-localize with RAD51 on asynapsed chromosomes in mouse spermatocytes, suggesting it functions in meiotic recombination [19]. In *C*. *elegans*, *brc-1* and *brd-1* mutants have mild meiotic phenotypes consistent with a role in some aspect of meiotic recombination [9, 10]. However, the relationship between BRC-1-BRD-1 function in synapsis and recombination has not been explored.

Here, we assessed BRC-1 and BRD-1 dynamics in the *C*. *elegans* germ line. Surprisingly, BRC-1-BRD-1 localizes to the synaptonemal complex (SC), becomes concentrated onto chromosome regions upon crossover designation, and at late meiotic prophase is restricted to the short arm of each bivalent as defined by the single crossover site on *C*. *elegans* chromosomes. BRC-1 and BRD-1 are interdependent for localization to the SC and proper localization is dependent on meiotic recombination and chromosome synapsis. Further, our data suggest that the BRC-1-BRD-1 complex promotes homologous recombination under meiotic dysfunction by stabilizing the RAD-51 filament and altering the patterning of crossovers. Similar findings are reported by Janisiw et al. in the accompanying paper.

## Results

### GFP::BRC-1 and BRD-1::GFP are expressed in embryos and the germ line

To examine BRC-1 and BRD-1 expression and localization in *C*. *elegans*, we engineered GFP::BRC-1 and BRD-1::GFP fusions at the endogenous loci using CRISPR-Cas9 [20]. *brc-1* and *brd-1* mutants have low levels of embryonic lethality, produce slightly elevated levels of male progeny (*X0*), a readout of *X* chromosome nondisjunction, and display sensitivity to **γ**-irradiation (IR) [10]. Worms expressing these fusions as the only source of BRC-1 or BRD-1 produced wild-type levels of viable progeny and males, and were not sensitive to IR (S1A–S1C Fig), suggesting that the fusions are fully functional.

**Fig 1:**
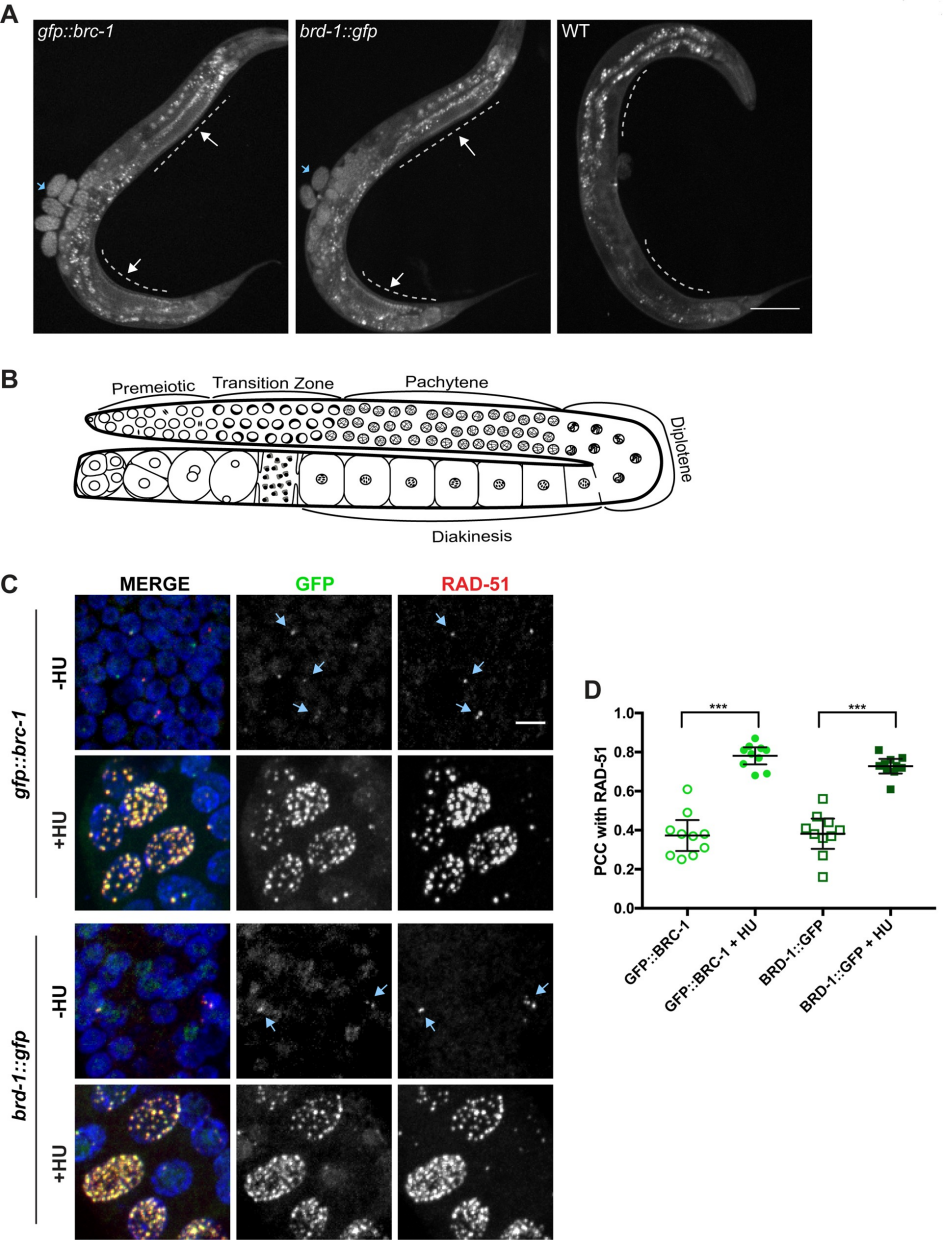
GFP::BRC-1 and BRD-1::GFP are expressed in the germ line and respond to stalled/collapsed replication forks. A) GFP fluorescence of whole worms expressing GFP::BRC-1, BRD-1::GFP, or no GFP (WT). Dashed line denotes germ line with arrows to indicate GFP fluorescence; blue arrows denote GFP signal in embryos; gut granules auto-fluoresce. Scale bar = 100μm. B) Schematic of the spatiotemporal organization of the hermaphrodite germ line with meiotic stages indicated. C) Proliferating germ cells (premeiotic) expressing GFP::BRC-1 or BRD-1::GFP (green), stained with antibodies against RAD-51 (red), and counterstained with DAPI (blue) in the absence (-HU) and presence of 5mM hydroxyurea (+HU). Blue arrows denote co-localization between GFP and RAD-51. Images are projections through half of the gonad. Scale bar = 5μm. D) Pearson’s Correlation Coefficient (PCC) measurements between RAD-51 and GFP::BRC-1 or BRD-1::GFP in the absence and presence of HU. 95% Confidence Intervals are shown. *** p< 0.0001, Mann-Whitney test; n = 10 nuclei.

We monitored the localization of GFP::BRC-1 and BRD-1::GFP by live cell imaging. In whole worms, GFP fluorescence was observed in embryos and in the germ line, with very little signal in the soma (note auto-fluorescence of gut granules also observed in wild type; Fig 1A). Immunoblots of whole worm extracts of *gfp*::*brc-1; fog-2*, which are true females [21] and therefore do not contain embryos, compared to self-fertilizing *gfp*::*brc-1* hermaphrodites containing embryos, revealed that <10% of the GFP::BRC-1 signal is due to expression in embryos (S1E Fig). Thus, BRC-1 and BRD-1 are expressed predominantly in the germ line.

### BRC-1-BRD-1 and RAD-51 become concentrated in foci upon replication stress

The *C*. *elegans* germ line is arranged in a spatio-temporal gradient, with proliferating germ cells (premeiotic) and all stages of meiosis arrayed from the distal to proximal end [22] (Fig 1B). We first focused on the premeiotic zone, where germ cells are mitotically proliferating. GFP::BRC-1 and BRD-1::GFP were observed diffusely throughout the nucleus, with occasional foci that partially co-localized with the recombinase RAD-51 (Fig 1C and 1D). In mammalian cells RAD51 marks stalled/collapsed replication forks [23], and BRCA1/BRC-1 has been implicated in repair of damaged forks in both mammals and *C*. *elegans* [14, 24]. To determine whether BRC-1-BRD-1 responds to stalled/collapsed replication forks, we treated worms with the ribonucleotide reductase inhibitor, hydroxyurea (HU). HU slows replication causing fork stalling and collapse, and cell cycle arrest leading to enlarged nuclei [23, 25]. GFP::BRC-1 and BRD-1::GFP fluorescence became enriched in many foci following exposure to HU, and these exhibited substantial co-localization with RAD-51 (Fig 1C and 1D). Consistent with a role in resolving collapsed replication forks, both *brc-1* and *brd-1* mutants were sensitive to HU as measured by embryonic lethality (S1D Fig). These results suggest that BRC-1-BRD-1 responds to replication stress and concentrates in foci where it co-localizes with RAD-51, presumably to resolve stalled/collapsed replication forks.

### BRC-1 and BRD-1 localize to the SC and concentrate to the short arm of the bivalent during meiotic prophase

**Fig 2:**
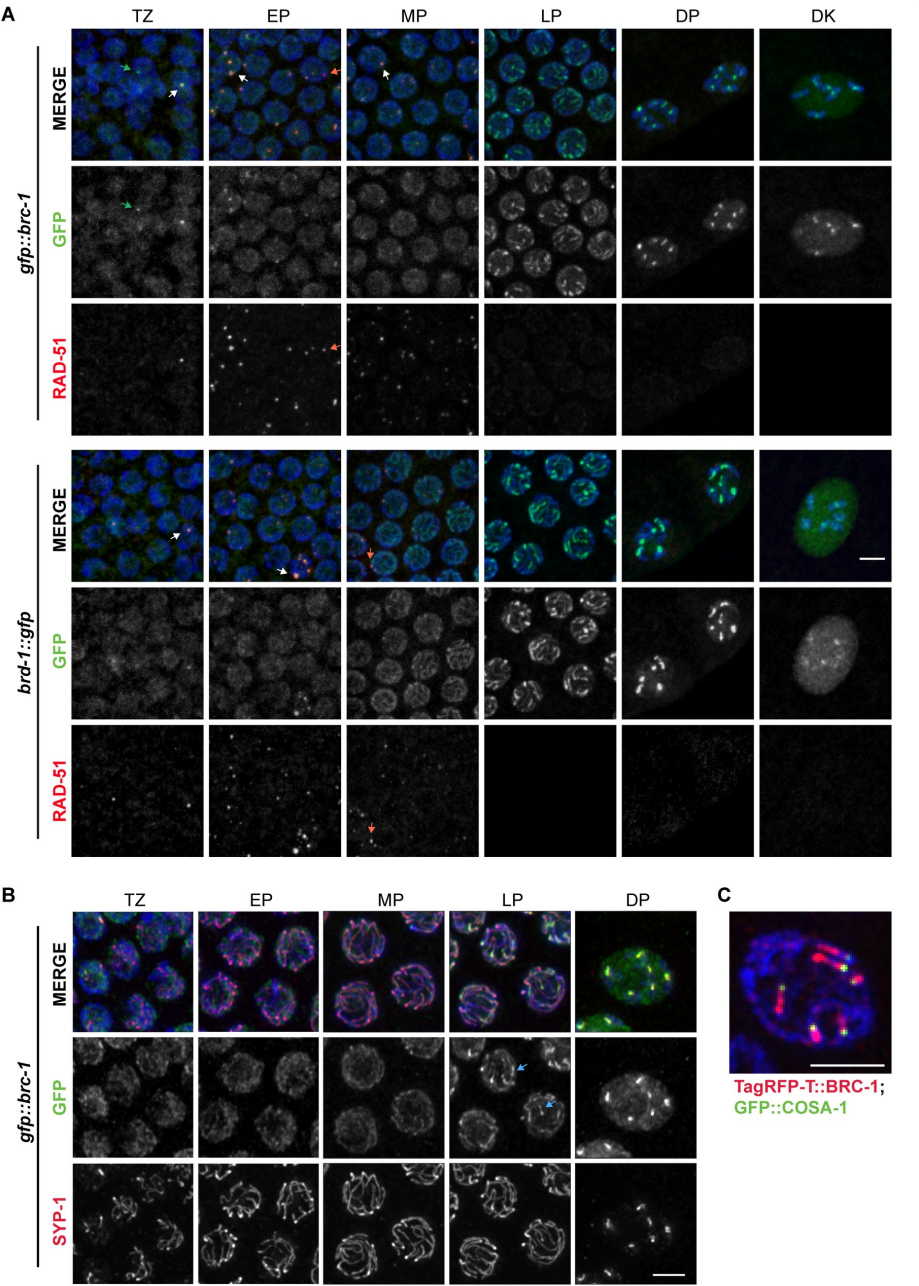
GFP::BRC-1 and BRD-1::GFP localize to the SC in meiotic prophase. A) Nuclei from indicated meiotic stages stained with RAD-51 antibodies (red), DAPI (blue) and imaged for GFP fluorescence (green). White arrows demark foci positive for both GFP fluorescence and RAD-51 signal; green arrows demark foci containing GFP but not RAD-51; red arrows demark foci containing only RAD-51. Scale bar = 5 μm. B) Co-localization between GFP::BRC-1 (green) and SC central component SYP-1 (red) by antibody staining; germ lines at indicated stages were counterstained with DAPI. Blue arrows at late pachytene show chromosomal regions where GFP::BRC-1 concentrates before SYP-1. Scale bar = 2 μm. C) TagRFP-T::BRC-1 (red) and GFP::COSA-1 (green) at late pachytene showing TagRFP-T::BRC-1 on one side of the GFP::COSA-1 focus, which marks the persumptive crossover. Scale bar = 2 μm. Images are projections through half of the gonad. TZ = transition zone; EP = early pachytene; MP = mid pachytene; LP = late pachytene; DP = diplotene; DK = diakinesis.

**Fig 3:**
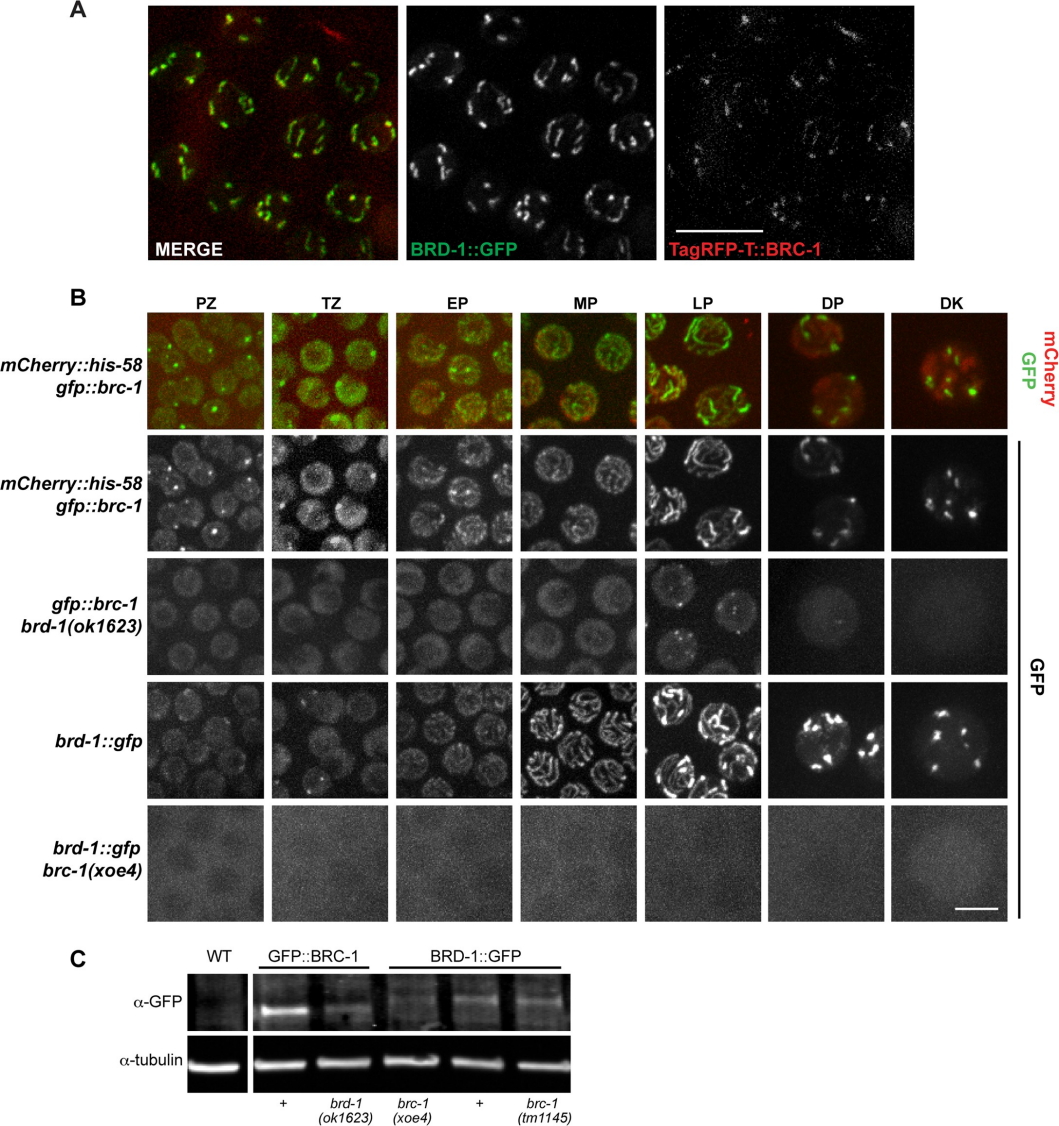
BRC-1 and BRD-1 are inter-dependent for localization. A) Co-localization between BRD-1::GFP (green) and TagRFP-T::BRC-1 (red) at late pachytene in live worms. Scale bar = 10 μm. B) Stills of germline nuclei from live worms expressing GFP::BRC-1 and mCherry::Histone H2B (*mCherry*::*his-58; gfp*::*brc-1*); merge and GFP channel; top two panels, respectively. GFP::BRC-1 expression in *brd-1(ok1623)* mutant at indicated meiotic stages. Bottom two panels show BRD-1::GFP localization in wild type and the *brc-1(xoe4)* mutant. Images are projections through half of the gonad. TZ = transition zone; EP = early pachytene; MP = mid pachytene; LP = late pachytene; DP = diplotene; DK = diakinesis. Scale bar = 5 μm. C) Immunoblot of whole worm extracts from indicated worms probed with anti-GFP and α-tubulin antibodies. Lane 1 = N2: wild type; Lane 2 = JEL515: *gfp*::*brc-1;* Lane 3 = JEL520: *gfp*::*brc-1 brd-1(ok1623);* Lane 4 = JEL744: *brc-1(xoe4) brd-1*::*gfp*; Lane 5 = JEL657: *brd-1*::*gfp*; Lane 6 = JEL678: *brc-1(tm1145) brd-1*::*gfp*.

**Fig 4:**
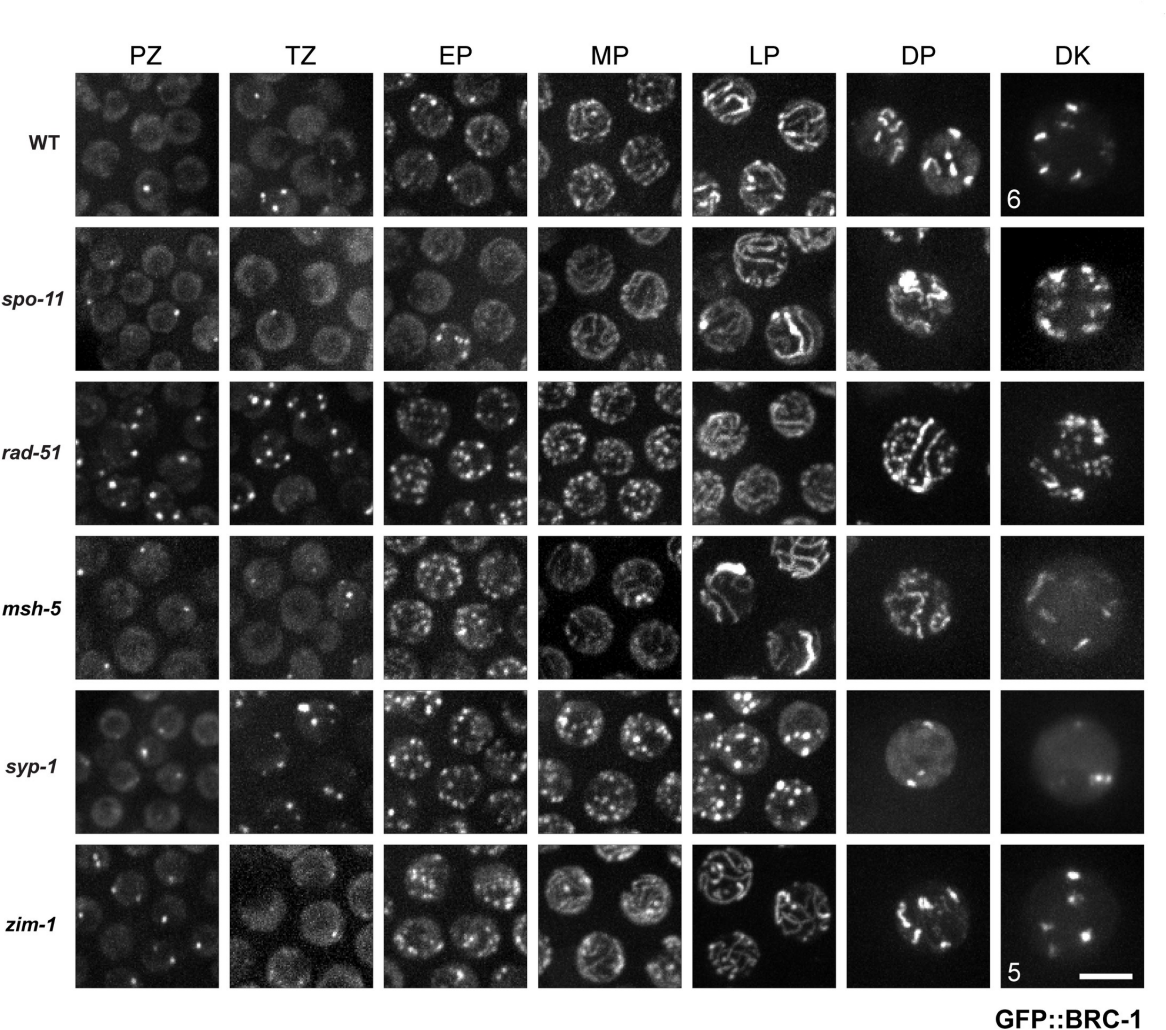
GFP::BRC-1 localization is perturbed when either meiotic recombination or chromosome synapsis is impaired. High-magnification images of live *C*. *elegans* expressing GFP::BRC-1 from the indicated genetic backgrounds and gonad region (PZ = Proliferative Zone, TZ = Transition Zone, EP = Early Pachytene, MP = Mid Pachytene, LP = Late Pachytene, DP = Diplotene, DK = Diakinesis). In wild-type worms GFP::BRC-1 localizes to chromatin and in a small number of foci in the proliferative and transition zones. GFP::BRC-1 localizes to long tracks corresponding to the SC in mid-pachytene. In late pachytene, GFP::BRC-1 becomes condensed to one side of each chromosome, demarcating what will become the short arms of the six bivalents in diakinesis (WT = 6). This localization pattern is perturbed when synapsis and crossover formation are disrupted. A *zim-1* diakinesis nucleus with 5 GFP::BRC-1 short stretches is shown. Images are projections through half of the gonad. Scale bar = 5 μm.

In early meiotic prophase (transition zone/early pachytene), GFP::BRC-1 and BRD-1::GFP direct fluorescence were observed diffusely on chromatin and in foci (Fig 2A). These foci partially overlapped with RAD-51, which marks meiotic DSBs [26]. We noticed that the relative intensity of the foci was weaker in fixed versus live imaging (see Figs 3 and 4), suggesting that these foci were sensitive to fixation conditions. Beginning at mid-pachytene, GFP::BRC-1 and BRD-1::GFP were observed in tracks along the entire chromosome length, and then concentrated to a portion of each chromosome at late pachytene (Fig 2A). In diplotene and diakinesis, GFP::BRC-1 and BRD-1::GFP were further restricted to six short stretches on the six pairs of homologous chromosomes (Fig 2A). As oocytes continued to mature, GFP::BRC-1 and BRD-1::GFP were disassembled from chromosomes in an asynchronous manner, with some chromosomes losing signal before others. Thus, in diakinesis nuclei we did not always observe six stretches of fluorescence, and the fluorescence intensity varied between chromosomes.

The concentration of BRC-1-BRD-1 into tracks at mid-pachytene suggested that the complex localized to the SC. To investigate this, we co-stained with antibodies against GFP and the SC central region component, SYP-1 [27]. Homologous chromosomes begin synapsing early in meiotic prophase (29); however, GFP::BRC-1 was not observed on tracks until after the SC appeared to be fully formed (Fig 2B). Interestingly, the concentration of GFP::BRC-1 to a portion of each chromosome preceded the relocalization of SYP-1 to the short arm of the bivalent (arrows in late pachytene images of GFP::BRC-1; Fig 2B). As the SC reorganizes as a consequence of crossover maturation [28], we examined worms co-expressing TagRFP-T::BRC-1 (TagRFP-T is a RFP variant with improved photostability [20, 29]) and GFP::COSA-1, a cyclin related protein that marks presumptive crossover sites [30]. TagRFP-T::BRC-1 also appeared to be fully functional (S1A–S1C Fig), although the fluorescent signal was weaker than GFP, and could only be detected in mid-late pachytene through diakinesis. GFP::COSA-1 was observed at one end of each TagRFP-T::BRC-1 stretch (Fig 2C). Thus, BRC-1 and BRD-1 localize to the SC and are redistributed concomitant with crossover designation, suggesting that BRC-1-BRD-1 functions in one or more aspects of meiotic recombination within the context of the SC.

### BRC-1 and BRD-1 are interdependent for localization

In both mammalian cells and *C*. *elegans*, BRCA1/BRC-1 and BARD1/BRD-1 form a stable complex [2, 31]. To probe the relationship between *C*. *elegans* BRC-1 and BRD-1 *in vivo*, we imaged live worms heterozygous for both TagRFP-T::BRC-1 and BRD-1::GFP (*brc-1* and *brd-1* are linked). In the heterozygous state the TagRFP-T signal could only be detected at late pachytene through early diakinesis when BRC-1 and BRD-1 are concentrated on short tracks. The TagRFP-T and GFP signals overlapped, suggesting that BRC-1 and BRD-1 are localized together on the SC (Fig 3A).

To examine localization dependencies between BRC-1 and BRD-1 in *C*. *elegans* germ cells, we monitored GFP::BRC-1 and BRD-1::GFP in the corresponding *brd-1(ok1623)* and *brc-1(xoe4)* null mutant backgrounds by live cell imaging. In the absence of BRD-1 we observed diffuse GFP::BRC-1 fluorescence within the nucleoplasm from proliferative zone to mid-pachytene, with no evidence of tracks (Fig 3B). In late pachytene, weak GFP::BRC-1 foci were observed; however, in diplotene and diakinesis only a diffuse nucleoplasmic signal was detected, with no concentrated regions of GFP::BRC-1. This result suggests that BRD-1 is required for the correct localization of BRC-1 in meiotic cells. In worms harboring a null allele of *brc-1*, BRD-1::GFP was largely cytosolic, except at diakinesis where it was observed in the nucleoplasm. Analysis of steady state protein levels by immunoblot revealed that BRC-1 and BRD-1 were present, albeit at reduced levels, in the absence of the other partner (in *brc-1(xoe4)*, BRD-1::GFP = 60% of wild-type levels; in *brd-1(ok1623)*, GFP::BRC-1 = 50% of wild-type levels; Fig 3C). Thus, BRC-1 and BRD-1 are mutually dependent for localization to meiotic chromosomes.

### Impairment of either meiotic recombination or synaptonemal complex formation alters GFP::BRC-1 localization

To provide insight into the relationship between BRC-1-BRD-1 and the progression of meiotic recombination, we monitored the localization of GFP::BRC-1 in mutants that impair different steps of meiotic recombination: *spo-11* mutants are unable to form meiotic DSBs [32, 33], *rad-51* mutants are blocked prior to strand invasion [34–36], and *msh-5* mutants fail to form crossovers [37, 38]. In live *spo-11* mutants, we observed many fewer GFP::BRC-1 foci in transition zone and early pachytene compared to WT (TZ: 1.29±0.12 vs. 0.18±0.05; EP: 4.61±0.36 vs. 0.91±0.22 foci/nucleus in WT and *spo-11*, respectively; p <0.0001; S2A Fig). At mid-pachytene GFP::BRC-1 was observed in tracks in the *spo-11* mutant similar to wild type, as synapsis occurs in the absence of meiotic DSB formation in *C*. *elegans* [32] (Fig 4). In late pachytene, GFP::BRC-1 fluorescence did not concentrate on a portion of each chromosome pair nor retract to the short arm of the bivalent as in wild type, consistent with these events being dependent on meiotic recombination. However, in 20.23±1.78% of nuclei (n = 4 germ lines) there was enrichment of GFP::BRC-1 on one or sometimes two tracks, in addition to weak staining on other tracks. This is similar to what has been previously reported for synapsis markers, including the phosphorylated form of SYP-4 [39–41], and likely represents *spo-11*-independent lesions capable of recruiting meiotic DNA repair components and altering SC properties. Consistent with this, we observed GFP::BRC-1 enrichment on the phospho-SYP-4-marked chromosome in *spo-11* mutants (S2B Fig). However, GFP::BRC-1 did not retract to chromosome subdomains as in wild type in diplotene and diakinesis, suggesting that the relocalization of BRC-1-BRD-1 is dependent on formation of meiotic DSBs. As expected, BRD-1::GFP was observed in a similar pattern to GFP::BRC-1 in *spo-11* mutants throughout meiotic prophase (S2C Fig).

Following DSB formation and processing, RAD-51 is loaded onto resected single-stranded DNA and facilitates strand exchange [36]. GFP::BRC-1 localization was altered in the *rad-51* mutant (Fig 4). Significantly increased levels of GFP::BRC-1 foci were observed throughout the germ line. In the proliferative zone, wild type had 0.55±0.04, while *rad-51* had 0.96±0.10 foci per nucleus (S2A Fig). These most likely represent concentration of GFP::BRC-1 at stalled/collapsed replication forks. In transition zone, wild type had 1.29±0.12, while *rad-51* had 3.98±0.31 foci/nucleus, and this was further increased in early pachytene (WT: 4.6±0.4 vs. *rad-51*: 13.3±0.7; S2A Fig). These foci presumably represent resected meiotic DSBs that fail to undergo strand invasion in the absence of RAD-51, as they cannot be solely accounted for by the elevated foci observed in proliferating cells. Track-like structures were not observed until late pachytene in the absence of RAD-51. The punctate nature of GFP::BRC-1 was particularly pronounced in diplotene and diakinesis, with no clear concentration to six regions. This is consistent with the disorganized chromatin masses observed in *rad-51* diakinesis nuclei [35], and suggests that RAD-51 is required for the proper organization and retraction of GFP::BRC-1.

In *msh-5* mutants, GFP::BRC-1 appeared similar to wild type from the proliferative zone to mid pachytene, localizing in the nucleoplasm and concentrating in foci before converging on tracks (Fig 4; S2A Fig). Similar to *spo-11*, 26.27±2.25% of *msh-5* late pachytene nuclei (n = 4 germ lines) contained enrichment of GFP::BRC-1 on one or occasionally two chromosomes. In diplotene, GFP::BRC-1 was observed in long tracks, with no evidence of retraction. The presence of more than six stretches of GFP::BRC-1 in diakinesis suggests that BRC-1 remains associated with the univalents in *msh-5* mutants. Taken together, our data suggest that GFP::BRC-1 localizes to the SC and its retraction to the short arm of the bivalent is dependent on processing of meiotic DSBs into crossovers.

We also examined localization of GFP::BRC-1 when synapsis is blocked by mutation of a component of the central region of the SC, *syp-1* [27]. GFP::BRC-1 in *syp-1* looked similar to wild type in proliferating germ cells (Fig 4). However, as cells entered meiosis GFP::BRC-1 was observed in many foci (in TZ, WT: 1.29±0.12 vs. *syp-1*: 7.29±0.36 foci/nucleus; S2A Fig). The number of foci increased through early and mid pachytene but GFP::BRC-1 never attained nuclear track staining, supporting a dependency on the SC for track localization. Similarly, the GFP::BRC-1 signal did not localize to sub-regions of condensed (DP and DK) chromosomes, but rather was found in a small number of nuclear foci. Thus, GFP::BRC-1 localization to tracks is dependent on SC formation.

To examine localization under conditions where a subset of chromosomes fail to synapse and recombine, we monitored GFP::BRC-1 localization in the *zim-1* mutant, in which chromosomes *II* and *III* cannot synapse [42]. In transition zone and early pachytene, GFP::BRC-1 was observed in many foci in the *zim-1* mutant, similar to the *syp-1* mutant (TZ: WT: 1.29±0.12 vs. *zim-1*: 4.5±0.36 foci/nucleus; Fig 4; S2A Fig). However, as meiosis progressed GFP::BRC-1 was observed on tracks that condensed to the short arm of the bivalent on multiple chromosomes. Many times we observed more than four stretches of GFP::BRC-1 fluorescence at diplotene/diakinesis (Fig 4), suggesting that there are more than four chiasmata in the *zim-1* mutant. We address the role of BRC-1 in chiasmata formation in the *zim-1* mutant below.

### The BRC-1-BRD-1 complex is important when chromosome synapsis and crossover formation are perturbed

Given the association of GFP::BRC-1 and BRD-1::GFP with the SC (Fig 4), we next examined the functional consequence of removing BRC-1-BRD-1 when synapsis is perturbed. For these studies we focused on the *zim-1* mutant, as the appearance of more than four short tracks of GFP::BRC-1 at diplotene/diakinesis (Fig 4) suggested that these BRC-1-BRD-1-associated regions were altered in the absence of *zim-1*. Additionally, unlike mutants such as *syp-1* that result in a complete failure in synapsis and therefore 95% embryonic lethality [27], loss of ZIM-1 results in 73.9% inviable progeny [42], allowing us to determine whether removal of BRC-1-BRD-1 enhances embryonic lethality.

**Fig 5:**
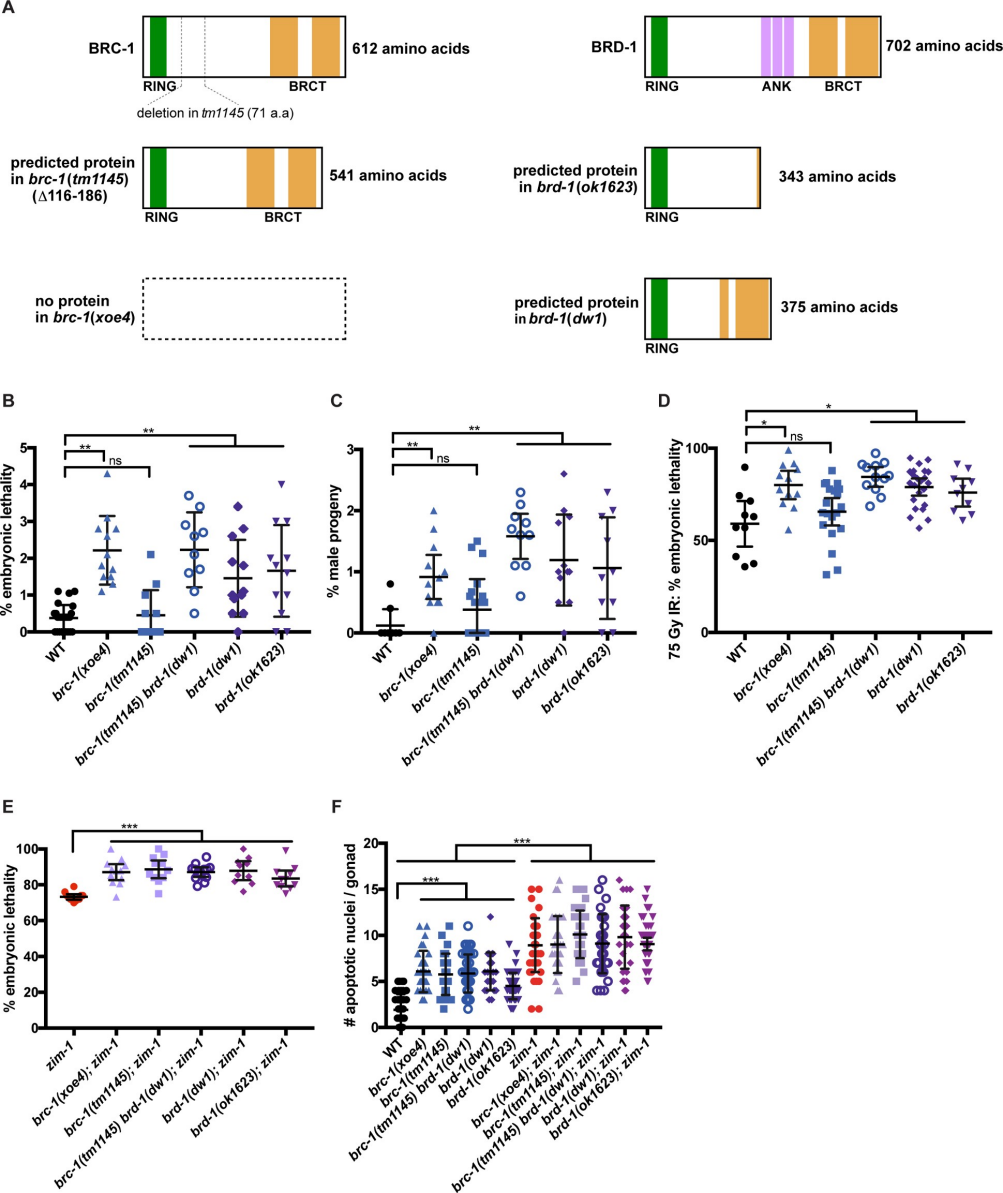
Differential effect of *brc-1* and *brd-1* alleles on the DNA damage response and meiosis. A) Cartoon of predicted proteins produced from the different *brc-1* and *brd-1* mutant alleles based on cDNA analysis (see also S3A Fig). RING (green), BRCT (gold) and Ankyrin (ANK; purple) domains are indicated. B) % embryonic lethality of *brc-1* and *brd-1* mutants; numbers of animals scored: WT = 26; *brc-1(xoe4)* = 12; *brc-1(tm1145)* = 12; *brc-1(tm1145) brd-1(dw1*) = 10; *brd-1(dw1)* = 12; *brd-1(ok1623)* = 12. C) % male progeny produced from *brc-1* and *brd-1* mutants; numbers of animals scored: WT = 10; *brc-1(xoe4)* = 12; *brc-1(tm1145)* = 22; *brc-1(tm1145) brd-1(dw1*) = 10; *brd-1(dw1)* = 12; *brd-1(ok1623)* = 10. D) % embryonic lethality following 75 Gy IR; numbers of animals scored: WT = 10; *brc-1(xoe4)* = 12; *brc-1(tm1145)* = 20; *brc-1(tm1145) brd-1(dw1*) = 12; *brd-1(dw1)* = 23; *brd-1(ok1623)* = 10. E) % embryonic lethality of *zim-1* in the presence and absence of BRC-1-BRD-1. Numbers of animals scored: *zim-1* = 12; *brc-1(xoe4); zim-1* = 12; *brc-1(tm1145); zim-1* = 22; *brc-1(tm1145) brd-1(dw1*); *zim-1* = 10; *brd-1(dw1); zim-1* = 12; *brd-1(ok1623); zim-1* = 10. The genetic interaction between *brc-1/brd-1* and *zim-1* is significant by a one-way ANOVA (p<0.0001). F) Number of apoptotic nuclei/gonad as scored by acridine orange. Numbers of gonads scored: WT = 43; *brc-1(xoe4)* = 29; *brc-1(tm1145)* = 25; *brc-1(tm1145) brd-1(dw1*) = 41; *brd-1(dw1)* = 24; *brd-1(ok1623)* = 50; *zim-1* = 42; *brc-1(xoe4); zim-1* = 30; *brc-1(tm1145); zim-1* = 36; *brc-1(tm1145) brd-1(dw1*); *zim-1* = 30; *brd-1(dw1); zim-1* = 31; *brd-1(ok1623); zim-1* = 46. 95% Confidence Intervals are shown. Statistical comparisons by Mann-Whitney: * p<0.05; ** p<0.001; *** p<0.0001; ns = not significant.

In the course of our experiments we discovered that strain DW102 [31] harbors mutations in both *brc-1* and *brd-1*; sequence analysis revealed that *brc-1(tm1145)* is an in-frame deletion, removing 71 amino acids (116–186) C-terminal to the predicted RING domain, which in the mammalian ortholog is responsible for E3 ubiquitin ligase activity and dimerization with BARD1 [3, 43, 44] (Fig 5A). The *brd-1* mutation in DW102 is identical to *brd-1(dw1)* [31]; cDNA analysis revealed that the mutation results in the use of an alternative splice site to generate a protein missing 327 amino acids, leaving the RING domain intact (Fig 5A and S3A Fig). To discern the contributions of BRC-1 and BRD-1 we used CRISPR-Cas9 to generate a complete deletion of BRC-1, *brc-1(xoe4)* (Fig 5A and S3A Fig). We also examined the *brc-1(tm1145)* and *brd-1(dw1)* single mutants, the *brc-1(tm1145) brd-1(dw1)* double mutant and *brd-1(ok1623)*, which results in the removal of 359 amino acids C terminal of the RING domain (Fig 5A and S3A Fig). As expected, *brc-1(xoe4)*, *brd-1(dw1)*, *brc-1(tm1145) brd-1(dw1)*, and *brd-1(ok1623)* displayed slightly elevated embryonic lethality (Fig 5B), male progeny (Fig 5C), and IR sensitivity (Fig 5D). On the other hand, *brc-1(tm1145)* was not statistically different from wild type for embryonic lethality, production of male progeny or IR sensitivity, suggesting that this allele is not a null mutation (Fig 5B–5D). Consistent with this, BRD-1::GFP was stable (Fig 3C) and localized similarly to wild type in the *brc-1(tm1145)* mutant background (S3B Fig).

In contrast to the differential impact of the alleles on embryonic lethality, male progeny, and IR sensitivity, loss of *zim-1* in any of the *brc-1* or *brd-1* mutants resulted in enhanced embryonic lethality compared to the single *zim-1* mutant (p<0.0001; Fig 5E). These results suggest that the region C-terminal to the BRC-1 RING domain, which is deleted in *brc-1(tm1145)*, is important for promoting embryonic viability when chromosome pairing and synapsis are perturbed.

To determine the nature of the enhanced embryonic lethality of *zim-1* mutants when BRC-1-BRD-1 is impaired, we first monitored germline apoptosis. Apoptosis is an output of checkpoint signaling and is important for culling defective germ cells [45–47]. Previous studies had established that both *brc-1* [9] and *zim-1* [48] have elevated checkpoint-dependent germline apoptosis. We found that all *brc-1* and *brd-1* alleles, including *brc-1*(*tm1145)*, had elevated apoptosis (Fig 5F). Loss of *zim-1* resulted in higher levels of apoptosis than *brc-1* and *brd-1* mutants; however, the levels of apoptosis in the double *brc-1; zim-1* and *brd-1; zim-1* mutants were not significantly different than *zim-1* alone. We also analyzed SUN-1 phosphorylated on Serine12 (Sun-1 S12P), which is dephosphorylated following establishment of the obligate crossover, and serves as a readout of meiotic progression [49]. Loss of ZIM-1 resulted in persistent SUN-1 S12P, which was unaltered in the absence of BRC-1 (S3C Fig). These results suggest that BRC-1-BRD-1 does not function in known signaling pathways responsible for monitoring unrepaired DSBs or crossovers leading to apoptosis or cell cycle delay.

**Fig 6:**
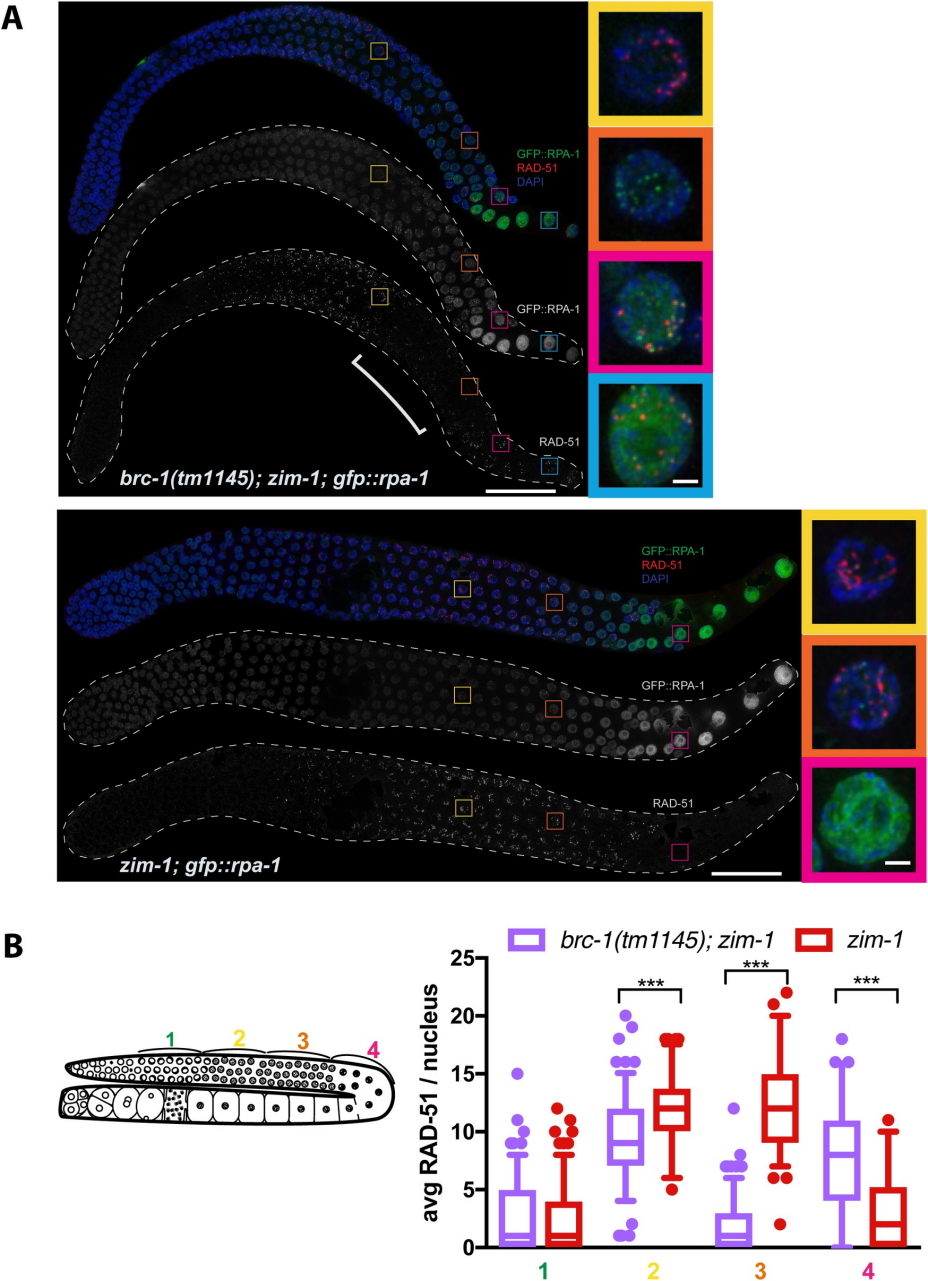
Inactivation of BRC-1-BRD-1 alters the pattern of RAD-51 foci in the *zim-1* mutant. A) Dissected germ lines from *brc-1(tm1145); zim-1; gfp*::*rpa-1* and *zim-1; gfp*::*rpa-1* worms stained with anti-RAD-51 (red), counterstained with DAPI (blue) imagined for GFP::RPA-1 fluorescence (green). Scale bar = 20 μm. Insets show selected nuclei from different regions of the germ line; bracket indicates RAD-51 “dark zone”. Images are projections through half of the gonad. A minimum of 3 germ lines were examined for each genotype. Scale bar = 1 μm. B) Schematic of germ line indicating zones for analysis of RAD-51 foci. Box whisker plots show average number of RAD-51 foci per nucleus in the different zones. Horizontal line of each box indicates the median, the top and bottom of the box indicates medians of upper and lower quartiles, lines extending above and below boxes indicate standard deviation and individual data points are outliers from 5–95%. Statistical comparisons by Mann-Whitney of *brc-1(tm1145); zim-1* versus *zim-1* in the different regions of the germ line; *** p<0.0001. A minimum of 3 germ lines were analyzed. Numbers of nuclei scored in each zone for *brc-1; zim-1*: 1 = 177; 2 = 138; 3 = 161; 4 = 61; *zim-1*: 1 = 159; 2 = 88; 3 = 103; 4 = 78.

We next monitored RAD-51 assembly/disassembly in the spatiotemporal organization of the germ line. Previous analyses revealed that *brc-1* and *brd-1* mutant hermaphrodites have elevated RAD-51 foci in late pachytene, suggesting that repair of a subset of meiotic DSBs is delayed in the absence of BRC-1-BRD-1 [9]; this was also observed in the *brc-1(tm1145) brd-1(dw1)* and *brd-1(ok1623)* mutants (S4A Fig). Further, blocking synapsis on some or all chromosomes results in elevated RAD-51 levels genome wide [26, 50], as observed in the *zim-1* mutant (Fig 6A and 6B). Surprisingly, *brc-1*; *zim-1* and *brd-1; zim-1* double mutants resulted in fewer RAD-51 at mid-late pachytene: RAD-51 foci appeared at similar levels compared to the *zim-1* single mutant early in meiotic prophase, but in the latter half of pachytene many fewer RAD-51 were detected on chromosomes (Fig 6A and 6B and S4B Fig). High levels of RAD-51 were observed again at the gonad bend, as nuclei exited pachytene and entered diplotene (Fig 6A and 6B and S4B Fig). Similar patterns were observed when BRC-1-BRD-1 was removed in other mutants that perturb synapsis (i.e., *syp-1*; S4B Fig). These results suggest that when synapsis and therefore crossover formation is impaired, BRC-1-BRD-1 plays a role in DSB formation, DNA end resection, RAD-51 loading, and/or stabilization of the RAD-51 filament in mid-late pachytene.

To differentiate between these possible meiotic functions of BRC-1-BRD-1, we analyzed the pattern of the single-stranded binding protein RPA-1 (GFP::RPA-1; [51]). RPA-1 binds resected ends prior to RAD-51 loading [52, 53] and is also associated with recombination events at a post-strand-exchange step, which can be observed in chromosome spreads [54]. In the *brc-1(tm1145); zim-1* germ line we observed an inverse pattern between RAD-51 and RPA-1 at mid-late pachytene: GFP::RPA-1 foci were prevalent in the region where RAD-51 foci were reduced (Fig 6A). In the *zim-1* single mutant, fewer GFP::RPA-1 foci were observed at this stage, while RAD-51 remained prevalent. We also observed very few RPA-1 foci at mid-late pachytene in wild type or *brc-1(tm1145) brd-1(dw1)* double mutant whole mount gonads (S4C Fig). These results suggest that BRC-1-BRD-1 is not required for DSB formation *per se* in this region of the germ line, as we observed an increase in GFP::RPA-1 foci, not a decrease as would be expected if BRC-1-BRD-1 mediates DSB formation. Additionally, this result argues against a role for BRC-1-BRD-1 in promoting resection as RPA-1 loads on exposed single stranded DNA [52]. Thus, at mid to late pachytene BRC-1-BRD-1 either facilitates the assembly of RAD-51 on new breaks, and/or stabilizes the RAD-51 filament.

### BRC-1-BRD-1 stabilizes the RAD-51 filament when crossover formation is impaired

**Fig 7:**
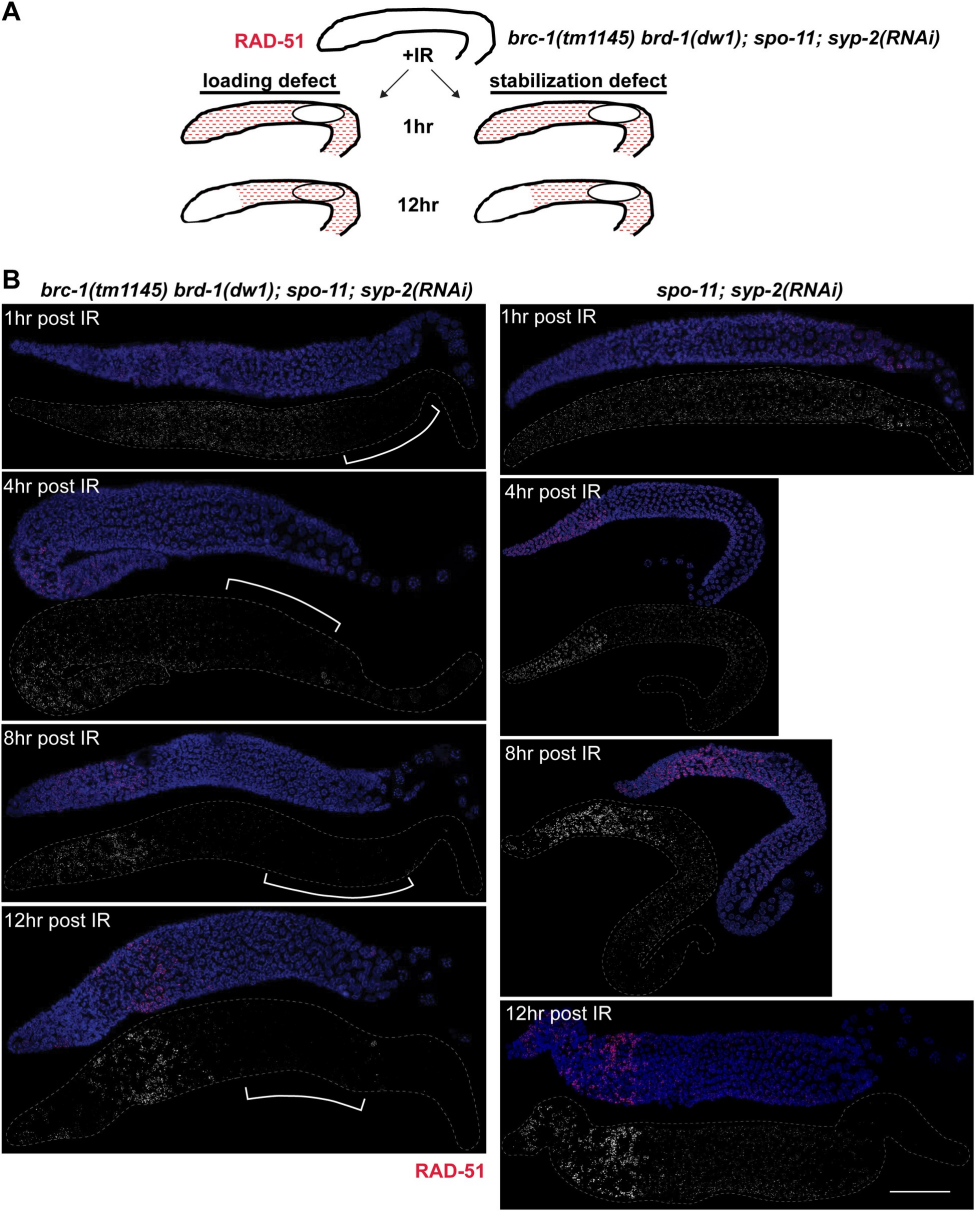
BRC-1 promotes the stability of RAD-51 filaments when crossover formation is impaired. A) Schematic of potential outcomes of IR-induced RAD-51 (red) over time in *brc-1(tm1145) brd-1(dw1); spo-11; syp-2*(RNAi) worms: a defect in RAD-51 loading would result in loss of the “dark zone” (circle) by 12 hrs (left), while a defect in RAD-51 stabilization would manifest in the maintenance of the dark zone over 12 hrs (right). B) Projections of whole germ lines at indicated times after IR treatment in *brc-1(tm1145) brd-1(dw1); spo-11; syp-2*(RNAi) (left) and *spo-11; syp-2*(RNAi) (right). DAPI (blue), RAD-51 (red). A minimum of 3 germ lines were imaged for each condition. Scale bar = 30 μm.

The lack of RAD-51 in mid to late pachytene in *brc-1; zim-1* and *brd-1; zim-1* mutants is reminiscent of the RAD-51 “dark zone” observed in the *rad-50* mutant following exposure to IR, which likely reflects a requirement for RAD-50 in loading RAD-51 at resected DSBs on meiotic chromosomes [55]. However, the distal boundary of the dark zone in the *brc-1; zim-1* double mutant is distinct from the *rad-50* mutant: the dark zone in *rad-50* extends from meiotic entry to late pachytene [55], while in the *brc-1; zim-1* and *brd-1; zim-1* mutants reduction in RAD-51 was limited to mid-late pachytene (Fig 6A and 6B and S4B Fig), suggesting that the nature of the dark zone is different in these mutant situations. If BRC-1-BRD-1 is required for loading RAD-51 on breaks in mid-late pachytene, then a time course analysis would reveal a diminution of the dark zone by twelve hours following IR exposure, as was observed for *rad-50* mutants (Fig 7A, loading defect on left) [55]. On the other hand, if BRC-1-BRD-1 was important for protecting RAD-51 from disassembly, then the dark zone should be maintained throughout the time course as RAD-51 would be disassembled as nuclei with pre-installed RAD-51 move through the mid-late pachytene region of the germ line (Fig 7A, stabilization defect on right). SPO-11 remains active under conditions where crossovers have not formed on all chromosomes [56, 57], making it difficult to distinguish a RAD-51 loading defect onto new breaks in this region of the germ line versus a defect in RAD-51 stability. Therefore, we performed these experiments in the *spo-11* mutant background [32], as IR will induce breaks uniformly in the germ line at a single point in time and as nuclei move through the germ line, no new breaks will be formed. *spo-11* is tightly linked to *zim-1*; consequently, we used RNAi against SYP-2, which in our hands is more efficient than *zim-1*(RNAi), to block synapsis and crossover formation. To that end, we exposed *spo-11* and *brc-1(tm1145) brd-1(dw1)*; *spo-11* mutants depleted for SYP-2 to 10 Gy of IR and examined RAD-51 over time. At one, four, eight, and twelve hours following IR, the dark zone was maintained in the absence of BRC-1-BRD-1 (Fig 7B). This result is consistent with the hypothesis that BRC-1-BRD-1 stabilizes the RAD-51 filament rather than facilitates loading of RAD-51 on new DSBs at mid-late pachytene.

### BRC-1-BRD-1 alters recombination patterning under meiotic dysfunction

**Fig 8:**
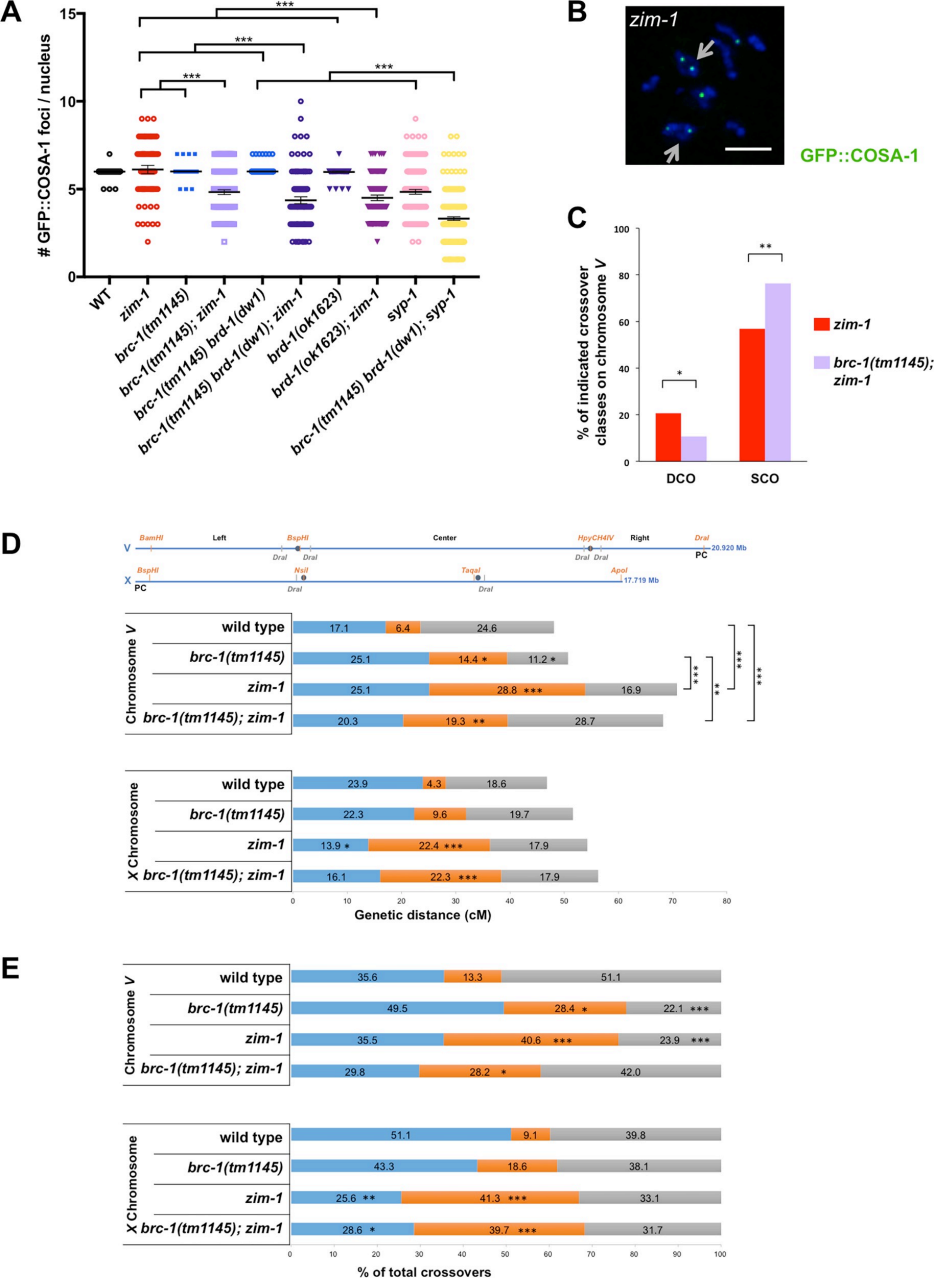
BRC-1-BRD-1 alters the crossover landscape when meiosis is impaired. A) Number of GFP::COSA-1 foci in mid-late pachytene in indicated mutants. Number of nuclei from a minimum of 4 germ lines scored: *gfp*::*cosa-1* = 458, *brc-1(tm1145) brd-1(dw1); gfp*::*cosa-1* = 815, *brc-1(tm1145) brd-1(dw1); zim-1; gfp*::*cosa-1* = 169*; brc-1(tm1145); gfp*::*cosa-1* = 235; *brc-1(tm1145); zim-1; gfp*::*cosa-1* = 255, *zim-1; gfp*::*cosa-1* = 120, *brd-1(ok1623); zim-1*; *gfp*::*cosa-1* = 164, *brd-1(ok1623); gfp*::*cosa-1* = 145, *syp-1; gfp*::*cosa-1* = 292, *brc-1(tm1145) brd-1(dw1); syp-1; gfp*::*cosa-1* = 487. The genetic interaction between *brc-1/brd-1* and *zim-1* is significant by a one-way ANOVA (*** p<0.0001). B) Diplotene *zim-1; gfp*::*cosa-1* nucleus showing ring-shaped chromosomes (arrows) and GFP::COSA-1 (green). Scale bar = 2 **μ**m. C) Percent recombinants for double crossover (DCO) [100 x DCO/(SCO+DCO+TCO)], p = 0.024, and single crossover (SCO) classes [100 x SCO/(SCO+DCO+TCO)], p = 0.0007, in *zim-1* and *brc-1(tm1145); zim-1* mutants. Statistical analyses were conducted using Fisher exact test on 2-by-2 contingency tables of DCO or SCO and total recombinants. D) Top: SNP markers: orange sites were analyzed from all embryos, grey sites were used to confirm potential double and triple COs; Bottom: CO frequency on chromosome *V* in wild type (n = 187), *brc-1(tm1145)* (n = 187), *zim-1* (n = 219) and *brc-1(tm1145); zim-1* (n = 192) mutants. CO frequency on the *X* chromosome in wild type (n = 188), *brc-1(tm1145)* (n = 188), *zim-1* (n = 223) and *brc-1(tm1145); zim-1* (n = 112) mutants. n = number of embryos analyzed per genotype. E) CO distribution among recombinants on chromosome *V* in wild type (n = 90), *brc-1(tm1145)* (n = 95), *zim-1* (n = 155) and *brc-1(tm1145); zim-1* (n = 131) and on the *X* chromosome in wild type (n = 88), *brc-1(tm1145)* (n = 97), *zim-1* (n = 121) and *brc-1(tm1145); zim-1* (n = 63). n = total number of COs per genotype; statistical analyses were conducted using χ^2^ on 2-by-2 contingency tables, * p<0.05; ** p<0.001; *** p<0.0001.

A subset of RAD-51 strand invasions are processed into crossovers, which are marked by CNTD1/COSA-1 [30, 58]. Given the reduction in RAD-51 in mid-late pachytene in *brc-1; zim-1* and *brd-1; zim-1* mutant hermaphrodites, we next analyzed crossover precursor formation in the various mutants. In *C*. *elegans*, each of the six chromosome pairs forms a single crossover; consequently, there are six COSA-1 foci in hermaphrodite germ cells at late pachytene [30] (Fig 8A). We also observed six COSA-1 foci in late pachytene nuclei in the *brc-1* and *brd-1* mutants (Fig 8A), indicating that breaks are efficiently processed into crossovers in the absence of BRC-1-BRD-1 in an otherwise wild-type worm. This is consistent with the presence of six bivalents at diakinesis and the low embryonic lethality of *brc-1* and *brd-1* [9, 10] (Fig 5B). In *zim-1* mutants we expected to observe four COSA-1 foci per nucleus, one on each of the four paired chromosomes, but not on the unpaired chromosomes *II* and *III*. Contrary to our expectations, *zim-1* had an average of 6.12±0.12 COSA-1 foci (χ^2^: p<0.005), with a very broad distribution ranging from 2 to 9 foci; such a wide distribution is never observed in wild type [30] (Fig 8A; S5 Fig). Inactivation of BRC-1 and/or BRD-1 in *zim-1* reduced the number of GFP::COSA-1 foci to a range of 4.3–4.8 in the various mutants, closer to expectations although still significantly different than expected (χ^2^: p<0.005), and the distribution remained broad (p <0.0001; Fig 8A). These results suggest that when crossovers are unable to form between some homologs, additional COSA-1-marked crossover precursors are generated, and some of these are dependent on BRC-1-BRD-1.

The higher than expected numbers of COSA-1 foci observed in *zim-1* mutants could reflect recombination intermediates that do not go on to form chiasmata (i.e., non-crossovers or inter-sister crossovers). Alternatively, COSA-1 could mark *bona fide* inter-homolog crossovers, such that some chromosomes have more than one chiasma, as has been observed in mutants where the *X* chromosomes fail to pair and synapse [50]. As these two possibilities are not mutually exclusive, the extra COSA-1 foci could be due to a combination of both recombination outcomes. To provide insight into the nature of the extra COSA-1 foci, we analyzed COSA-1 in *syp-1* mutants, where no chiasmata can form as all chromosomes fail to synapse, and found that there were on average 4.85±0.07 COSA-1 foci at late pachytene (Fig 8A; S5 Fig). These results suggest that under conditions of meiotic dysfunction when chromosomes are unable to pair/synapse, COSA-1 is recruited to recombination intermediates that are processed into non-crossovers and/or inter-sister crossovers. Similar numbers of COSA-1 foci, associated with MSH-5, were observed in *syp-3* mutants; high resolution cytological analyses indicated that these recombination sites are non-randomly distributed but with some abnormalities, consistent with the formation of nonproductive intermediates or inter-sister crossovers [59]. As with *zim-1* mutants, inactivation of BRC-1-BRD-1 in the *syp-1* mutant background led to fewer COSA-1 foci (Fig 8A; S5 Fig), suggesting that BRC-1-BRD-1 promotes COSA-1-associated recombination processing when chiasma formation is impaired.

To determine whether the extra COSA-1 foci on synapsed chromosomes could form chiasmata, we examined *zim-1* and *brc-1(tm1145); zim-1* diplotene/diakinesis nuclei, where chromosomes are individualized and cross-shaped structures indicative of crossovers between homologs can be observed. Consistent with the formation of extra chiasmata in the *zim-1* mutant background, we observed 52% of diplotene/diakinesis nuclei (n = 52) containing at least one ring-shaped structure, and six had two ring-shaped structures. The simplest interpretation is that there was a chiasma on each end of the chromosome pair (arrow; Fig 8B). This was reduced to 21% of diplotene/diakinesis nuclei (n = 43) containing ring-shaped chromosomes in the *brc-1(tm1145); zim-1* double mutant (*zim-1* vs. *brc-1(tm1145); zim-1*, p = 0.0028 Mann-Whitney). These results suggest that BRC-1-BRD-1 promotes chiasma formation when some chromosomes are unable to interact with their partner.

To examine genetic crossovers, we monitored linkage between SNP markers on chromosomes *V* and *X* in Bristol/Hawaiian hybrid strains to assess both crossover numbers and distribution. While inactivation of *brc-1* had no effect on crossover numbers on chromosome *V* (WT = 48.1cM; *brc-1* = 50.8cM), we observed an altered distribution compared to wild type (Fig 8D and 8E; S1 Table). In *C*. *elegans*, crossovers are enriched on the arms [28, 60–62]; in the *brc-1(tm1145)* mutant we observed a more even distribution, with more crossovers in the center and fewer on the right arm (Fig 8E; S1 Table). On the other hand, in *brc-1(tm1145)*, neither crossover frequency nor distribution were significantly different on the *X* chromosome (Fig 8D and 8E), which has an altered crossover landscape compared to the autosomes [63, 64].

We next monitored linkage between SNP markers in the *zim-1* and *brc-1(tm1145); zim-1* mutants. We observed a significant increase in the recombination map on chromosome *V* in *zim-1* (70.8cM), and multiple double crossovers were observed (Fig 8D; S1 Table). Extra crossovers were also observed on autosomes in worms unable to pair and synapse *X* chromosomes [50]. Inactivation of BRC-1 in the *zim-1* background resulted in significantly fewer double crossovers (DCOs) on chromosome *V* (p = 0.0242; Fig 8C, S1 Table), although the overall genetic map length was not significantly different compared to the *zim-1* single mutant (68.2cM; Fig 8D). This is most likely a consequence of an increase in the single crossover class (SCO; *zim-1* vs. *brc-1(tm1145); zim-1*, p = 0.0007; Fig 8C, S1 Table). On the *X* chromosome crossover frequency and distribution were altered in the center region in both *zim-1* and *brc-1(tm1145); zim-1* and in the left interval in *zim-1*; however, the overall map lengths were not statistically different between any of the strains.

*C*. *elegans* exhibits strong interference, which is the phenomenon that a crossover at one position on a chromosome decreases the probability of formation of a crossover nearby, resulting in a single crossover per chromosome [62]. Given the detection of DCOs on chromosome *V* in the *zim-1* and *brc-1(tm1145); zim-1* mutants, we calculated the interference ratio. While wild type and *brc-1* had absolute intereference of 1, as no double crossovers were observed, the *zim-1* mutant displayed reduced interference in the left-center and left-right intervals and negative interference in the center-right interval (Table 1). Inactivation of BRC-1 in the *zim-1* mutant restored positive interference in the center-right interval; however, this fell short of statistical significant (p = 0.064). In addition to the non-randomness in the number and position of crossovers, interference also operates on the level of chromatids such that a crossover between any two non-sister chromatids can affect the probability of those chromatids being involved in other crossovers [65]. Chromatid interference has been shown to occur in fungi, Drosophila, maize and humans [65–70]. Since we assayed single products of meiosis, the SCO class includes single crossovers as well as recombinants that are the result of three- or four-strand double crossovers, while only two strand-events can be detected as DCOs. The elevated numbers of SCOs and reduction in two-strand DCOs on chromosome *V* in the *brc-1(tm1145); zim-1* mutant compared to the *zim-1* single mutant (Fig 8C), suggest that there may be more three- and/or four-strand double crossovers when BRC-1 is inactivated. Thus, BRC-1 may counteract chromatid interference under meiotic dysfunction, such that more two-strand double crossovers occur. Taken together, the reduced number of COSA-1 foci and alteration in the genetic map in the *brc-1(tm1145); zim-1* mutant suggest that BRC-1-BRD-1 modifies recombination patterning under meiotic dysfunction.

**Table 1:** Crossover interference on chromosome *V*.

WT (V)	expected DCO	observed DCO	c.o.c.	interference
LC	0.011	0.00	0.00	1.00
CR	0.016	0.00	0.00	1.00
LR	0.042	0.00	0.00	1.00
*brc-1* (V)	expected DCO	observed DCO	c.o.c.	interference
LC	0.036	0.00	0.00	1.00
CR	0.016	0.00	0.00	1.00
LR	0.028	0.00	0.00	1.00
*zim-1*(V)	expected DCO	observed DCO	c.o.c.	interference
LC	0.072	0.055	0.75	0.25
CR	0.049	0.087	1.79	-0.79
LR	0.042	0.018	0.43	0.57
*brc-1;zim-1*(V)	expected DCO	observed DCO	c.o.c.	interference
LC	0.039	0.026	0.67	0.33
CR	0.055	0.031	0.57	0.43
LR	0.058	0.031	0.54	0.46

LC = left-center interval; CR = center-right interval; LR = left-right interval. DCO: double crossover; expected DCO: (crossover frequency at interval “A”) x (crossover frequency at interval “B”). c.o.c. (coefficient of coincidence) = actual DCO frequency/ expected DCO frequency; Interference = 1- c.o.c. See S1 Table for data used for calculations. Statistical analyses of interference using χ^2^ on 2-by-2 contingency tables of observed and expected DCOs [119], indicated that interference in the CR interval fell short of statistical significance between *zim-1* and *brc-1(tm1145); zim-1*, p = 0.064.

## Discussion

Here we show that *C*. *elegans* BRC-1 and BRD-1 orthologs localize to the SC and regulate recombination when meiosis is perturbed. Our results suggest that BRC-1-BRD-1 plays an important role in monitoring and modulating processing of meiotic DSBs into crossovers in the context of the specialized meiotic chromosome structure.

### BRC-1-BRD-1 undergoes dynamic localization that is coupled to crossover recombination

In mouse spermatocytes BRCA1 is associated with RAD51 and enriched on asynapsed regions of meiotic chromosomes, including the X-Y sex body [18, 19]. Here we show that *C*. *elegans* BRC-1 and BRD-1 partially co-localize with RAD-51 in early meiotic prophase, but become enriched on synapsed chromosomes as meiosis progresses, co-localizing with SYP-1, a SC central region component (Fig 2B). The enrichment of mammalian BRCA1 on asynapsed chromosomes versus BRC-1 on synapsed chromosomes in *C*. *elegans* most likely reflects alteration in the relationship between meiotic recombination and SC formation in these organisms. Meiotic chromosomes can pair and synapse in the absence of meiotic recombination in *C*. *elegans* [32], while these events are interdependent in mammals [15, 16]. The HORMAD axial components also show differences in chromosome association in mice and worms: in mice, HORMAD1 and HORMAD2 are enriched on asynapsed chromosomes [71, 72], while *C*. *elegans* HORMADS, HIM-3, HTP-1/2, and HTP-3, remain associated with synapsed chromosomes [73–76]. However, the function of HORMADs in preventing inter-sister recombination and in checkpoint signaling appears to be similar in these different organisms [77–82]. Thus, the association of BRC-1-BRD-1 to the SC in *C*. *elegans* is likely a consequence of the inter-relationship between SC formation and meiotic recombination in this organism and not due to different functions for this complex in worm versus mammalian meiosis.

Another difference between *C*. *elegans* and mammals is the nature of the kinetochore. *C*. *elegans* chromosomes are holocentric while in many organisms, including yeast and mice, chromosomes are monocentric. Holocentricity dictates that a single off-centered crossover is formed on each homolog pair to define the long and short arms necessary to ensure regulated sister chromatid cohesion release at meiosis I and II [60–62, 83]. Interestingly, BRC-1-BRD-1 becomes restricted to the short arm of the bivalent, as defined by the crossover site, and this precedes SC reorganization. While the absence of BRC-1-BRD-1 alone does not affect crossover formation on chromosome *V* and the *X* chromosome, it does have a subtle effect on the distribution of crossovers along chromosome *V* such that more occur in the middle of the chromosome (Fig 8D and 8E). The change in crossover distribution in *brc-1* mutants may contribute to the slightly increased nondisjunction observed in the absence of the BRC-1-BRD-1 complex.

We show that the concentration of BRC-1-BRD-1 to a portion of each chromosome track in late pachytene is dependent on meiotic DSB formation and processing into crossovers (*spo-11*, *rad-51* and *msh-5*; Fig 4). Interestingly, in both *spo-11* and *msh-5* mutants there are occasional chromosomal tracks in late pachytene, which are highly enriched for BRC-1. While synapsis markers also show occasional enrichment to single tracks in the absence of *spo-11*, and these partially overlap with BRC-1 (S2B Fig), no enrichment of synapsis markers is observed when crossover factors (i.e., *msh-5*, *cosa-1 or zhp-3*) are removed [39–41]. While it has been proposed that *spo-11*-independent lesions can recruit meiotic DNA repair components [39–41], the enrichment of BRC-1 in the absence of such crossover factors suggests that BRC-1-BRD-1 can respond to other repair intermediates in addition to those leading to inter-homolog crossovers. One possibility is that when inter-homolog crossover formation is blocked, DSBs are repaired through site-specific nucleases [84–86], a subset of which leads to the concentration of BRC-1-BRD-1 on chromosomes in late pachytene. This is also consistent with the observation that BRC-1 is maintained on chromosomes in *spo-11*, *rad-51* and *msh-5* mutants in diakinesis nuclei. Perhaps the failure to form interhomolog crossovers in these mutants leads to continued association of BRC-1-BRD-1 on chromosomes.

### BRC-1 and BRD-1 associate and are mutually dependent for localization to meiotic chromosomes

BRCA1 forms a potent E3 ubiquitin ligase only in complex with its partner BARD1 [2, 3]. Biochemical and structural studies have defined the RING domains and associated helices of these proteins as critical for catalytic activity and BRCA1-BARD1 interaction [43]. However, while the BRCA1-BARD1 heterodimer exhibits substantially greater E3 ligase activity *in vitro* than BRCA1 alone, only the BRCA1 RING domain interacts with the E2 for ubiquitin transfer, suggesting that BRCA1 is the critical subunit for E3 ubiquitin ligase activity [3, 87]. Structure-function analysis of the BARD1 RING domain suggests that BARD1 may serve to attenuate BRCA1 E3 ligase activity [88]. Interestingly, while the localization of BRC-1 and BRD-1 were interdependent (Fig 3B), BRD-1 appeared to be excluded from the nucleus in the absence of BRC-1, while BRC-1 was nucleoplasmic and formed foci in late pachytene in the absence of BRD-1 (Fig 3B). These differences may reflect the nature of the alleles examined: *brc-1(xoe4)* produces no protein, while the two *brd-1* alleles are predicted to produce truncated proteins, where the RING domain and associated helices remain intact (Fig 5A). In humans, BRCA1 nuclear localization signals in the middle of the protein can directly mediate nuclear import, or import can occur indirectly through interaction with BARD1 [89]. Thus, the truncated BRD-1 protein produced from the *brd-1(ok1623)* allele could associate with BRC-1 and facilitate nuclear localization of the albeit nonfunctional complex. Alternatively, *C*. *elegans* BRC-1 may be uniquely required for nuclear localization or retention, and in its absence BRD-1 cannot enter or be retained in the nucleus. The weak nucleoplasmic BRD-1 signal observed at the end of meiotic prophase in the absence of BRC-1 most likely reflects differences in the nuclear membrane as oogenesis proceeds [90, 91].

In addition to the N-terminal RING domains, both BRC-1 and BRD-1 contain long linker and phosphoprotein binding BRCA1 C-terminal (BRCT) domains. BRCT domains are phosphorylation-dependent interacting modules that have been implicated in tumor suppressor activity [92]. Interestingly, only BRD-1 contains Ankyrin (ANK) repeat interaction domains. Recent structural and functional analyses of the ANK domain in TONSL-MMS22L, a complex involved in homologous recombination, revealed that the ANK domain interacts with histone H4 tails [93]. The BARD1 ANK domains have a very similar fold [93], suggesting that BARD1 ANK domains may be important for association with chromatin. The predicted truncated proteins produced in the *brd-1* mutants, which behave as nulls (Fig 5 and S4 Fig), lack at least part of the BRCT domains and all of the ANK domains, suggesting that some combination of these domains are critical for BRD-1 function with respect to both DNA damage signaling and meiosis.

### BRC-1-BRD-1 function in meiotic recombination can be genetically separated from its established role in the DDR

It has long been appreciated that BRCA1-BARD1 mediates its tumor suppressor activity at least in part through regulating homologous recombination [6]. Given the importance of homologous recombination in repairing DSBs during meiosis, it is not surprising that removing BRC-1-BRD-1 impinges on meiotic recombination. Unexpectedly, we identified a small region C-terminal to the BRC-1 RING and associated helices as being important specifically for meiosis, suggesting that the function of BRC-1-BRD-1 in DNA damage response and meiosis are distinct. While containing no specific fold or homology, this region has several potential phosphorylation sites based on prediction algorithms that may mediate its interaction with key meiotic proteins.

BRCA1-BARD1 associates with the recombinase RAD51 in both mammals and *C*. *elegans* [19, 31, 94]. BRCA1 has also been shown to be required for the assembly of DNA damage induced RAD51 foci on chromatin [95], and this has been interpreted as a requirement for BRCA1 in RAD51 filament assembly. However, recent biochemical analyses using purified proteins found that BRCA1 is not required for RAD51 assembly on RPA coated single stranded DNA and instead promotes DNA strand invasion [94]. Further, a BARD1 mutant that cannot interact with RAD51 does not promote DNA strand invasion, and also does not form foci *in vivo*. Thus, it is likely that BRCA1-BARD1 is not required for RAD51 filament assembly *per se*. Our IR time course analysis of *C*. *elegans brc-1 brd-1* mutants is consistent with a function for this complex in stabilizing the RAD-51 filament. It is possible that similar to the mammalian complex, BRC-1-BRD-1 promotes RAD-51 strand invasion; however, *in vivo* the RAD-51 filament may be subject to disassembly by other proteins in the absence of BRC-1-BRD-1, which would not be recapitulated *in vitro*. One such protein is the FANCJ/DOG-1 helicase, which interacts with BRCA1 [96], and can disassemble RAD51 on ssDNA *in vitro* [97]. It is also likely that BRCA1-BARD1 plays multiple roles during homologous recombination and interacts with, and coordinates the activity, of many proteins, including RAD51, and these interactions are modulated under different conditions, including DNA damage, meiosis, meiotic dysfunction, as well as at different stages of the cell cycle. Consistent with this, Janisiw et al. found that BRC-1 associates with the pro-crossover factor MSH-5.

### BRCA1-BARD1 and meiotic checkpoint signaling

*brc-1* and *brd-1* mutants have very subtle defects in meiosis. These include low levels of X chromosome nondisjunction [10] (Fig 5C), a delay in repair of a subset of DSBs through the inter-sister pathway [9], and elevated heterologous recombination [12]. However, removing BRC-1-BRD-1 when meiosis is perturbed in mutants that impair chromosome pairing, synapsis and crossover recombination leads to enhanced meiotic dysfunction, including elevated embryonic lethality (Fig 5E), impaired RAD-51 stability (Fig 7), and alteration of COSA-1 numbers and the crossover landscape (Fig 8). These results suggest that BRC-1-BRD-1 plays a critical role in meiotic recombination when meiosis is impaired.

In both *C*. *elegans* and *Drosophila melanogaster*, preventing crossover formation on a subset of chromosomes leads to additional events on other chromosomes, and is referred to as the interchromosomal effect [50, 98–101]. There is also evidence in humans that Robertsonian translocations elicit the interchromosomal effect [102]. Our analyses of the *zim-1* mutant, where chromosomes *II* and *III* fail to recombine, revealed elevated COSA-1 foci genome wide and an increase in genetic crossovers on chromosome *V* (but not the *X* chromosome, Fig 8), consistent with the interchromosomal effect. Further, our data suggest that when meiosis is impaired as in *syp-1*, and perhaps *zim-1* mutants, COSA-1 can mark events that do not ultimately become interhomolog crossovers (see also [59]). Interestingly, removal of BRC-1-BRD-1 in *zim-1* and *syp-1* mutants decreased the number of COSA-1 foci. On the other hand, in the *brc-1(tm1145); zim-1* mutant we detected elevated levels of single crossovers but reduced levels of two-strand double crossovers on chromosome *V* compared to the *zim-1* single mutant, with no change in the overall map length (Fig 8D). One possibility to explain the observed COSA-1 and crossover pattern is that COSA-1 does not become enriched on a subset of crossovers in *brc-1; zim-1* mutants even though these events are dependent on the canonical meiotic crossover pathway, as observed in the *rtel-1* and *dyp-28* mutants [30, 103–105]. Alternatively, the extra crossovers that are not marked by COSA-1 in the absence of BRC-1-BRD-1 may be the result of activation of alternative crossover formation pathways. In either scenario, BRC-1, and presumably BRD-1, appear to dictate the patterning of crossovers among non-sister chromatids. As interference is mediated by meiotic chromosome structure [106], perhaps SC-associated BRC-1-BRD-1 counteracts chromatid interference in the context of meiotic dysfunction.

In conclusion, our results suggest that BRC-BRD-1 serves a critical role in monitoring the progression of meiotic recombination in the context of the SC when meiosis cannot proceed normally, suggesting that BRC-1-BRD-1 serves a checkpoint function. When crossover formation is blocked, BRC-1-BRD-1 stabilizes the RAD-51 filament and promotes processing of recombination intermediates marked by COSA-1. In this context, BRC-1-BRD-1 joins a growing list of proteins that monitor meiotic recombination to promote accurate chromosome segregation, including protein kinases and ubiquitin/SUMO E3 ligases [39, 56, 57, 107–110]. Future work will examine the relationship between BRC-1-BRD-1 and other meiotic checkpoint pathways and identify substrates of BRC-1-BRD-1-ubiquitination to understand how this complex modulates recombination under conditions when meiosis is perturbed.

## Materials and methods

### Generation of *gfp*::*brc-1*, *tag-rfp-T*::*brc-1*, *brd-1*::*gfp* and *brc-1(xoe4)*

Fluorescent protein knock-ins were generated using CRISPR-mediated homology dependent repair with self-excising cassette containing hygromycin resistant as selection [20]. The *brc-1(xoe4)* deletion allele was generated using Cas9-snRNPs and an single strand oligonucleotide repair template [111]. Cas9 protein was purchased from Innovative Genomics Institute, UC Berkeley. For a list of sgRNAs and repair templates refer to **S2 Table**. All CRISPR-generated lines were back crossed a minimum of three times, with the exception of JEL744 *brc-1(xoe4) brd-1*::*gfp*, which was only back crossed once.

### Genetics

*C*. *elegans* var. Bristol (N2), was used as the wild-type strain. Other strains used in this study are listed in **S3 Table**. Some nematode strains were provided by the Caenorhabditis Genetics Center, which is funded by the National Institutes of Health National Center for Research Resources. Strains were maintained at 20°C.

### Embryonic lethality and production of male progeny

Embryonic lethality in the absence or presence of 5mM hydroxyurea (HU) (16 hrs), or 75 Grays (Gy) of γ-irradiation (IR) from a ^137^Cs source, was determined over 3 days by counting eggs and hatched larvae 24 hr after removing the hermaphrodite and calculating percent as eggs/ (eggs + larvae); male progeny was assessed 48 hr after removing the hermaphrodite. A minimum of 10 worms were scored for each condition.

### Apoptosis assay

Acridine orange (AO) staining of apoptotic germ cells in WT (N2), *brc-1* and *brd-1* alleles as well as *zim-1* and corresponding double and triple mutants were performed as in [48]. Briefly, 0.5 ml of 50 mg/ml AO (Molecular Probes, Invitrogen; Carlsbad, CA) in M9 was added to 60-mm plates containing 48 hr post L4 worms and incubated at room temperature for 1 hr. Worms were transferred to new 60-mm plates, allowed to recover 15 min, and then mounted under coverslips in M9 on 3% agarose pads containing 1 mM tetramisole (Sigma-Aldrich; St. Louis). Apoptotic bodies were scored by fluorescence microscopy and DIC.

### Cytological analysis

Gonads were dissected and fixed with 1% paraformaldehyde in egg buffer plus 0.01% Tween20 for 5 min, freeze-cracked and post-fixed in either ice-cold 100% methanol for indirect immunofluorescence, or ice-cold 100% ethanol for direct fluorescence (GFP::BRC-1, TagRFP-T::BRC-1, BRD-1::GFP, GFP::RPA-1, GFP::COSA-1) for 1 min [112]. For staining with antibodies against phospho-SYP-4, gonads were dissected, freeze-cracked, incubated in 100% methanol for 1 min and post-fixed in 4% paraformaldehyde in PBS, 80mM HEPES(pH7.4), 0.8mM EDTA, 1.6mM MgS0_4_ for 30 min [40]. The following primary antibodies were used at the indicated dilutions: rabbit anti-RAD-51 (1:10,000; Catalog #29480002; Novus Biologicals, Littleton, CO), rabbit anti-GFP (1:500; NB600-308; Novus Biologicals, Littleton, CO), goat anti-SYP-1 (1:200; generously provided by Anne Villeneuve); rabbit anti-phospho-SYP-4 (1:100; [40]), and guinea pig anti-SUN-1 S12P (1:1,000; generously provided by Verena Jantsch). Secondary antibodies Alexa Fluor 594 donkey anti-rabbit IgG, Alexa Fluor 555 donkey anti-goat IgG, Alexa Fluor 488 donkey anti-rabbit IgG, Alexa Fluor 488 goat anti-guinea pig IgG from Life Technologies were used at 1:500 dilutions. DAPI (2μg/ml; Sigma-Aldrich) was used to counterstain DNA.

Collection of fixed images was performed using an API Delta Vision deconvolution microscope, a Nikon TiE inverted microscope stand equipped with an 60x, NA 1.49 objective lens and Andor Clara interline camera, or were captured on a spinning-disk module of an inverted objective fluorescence microscope [Marianas spinning-disk confocal (SDC) real-time 3D Confocal-TIRF (total internal reflection) microscope; Intelligent Imaging Innovations] equipped with an 63x, NA 1.46 objective lens using a Photometrics QuantiEM electron multiplying charge-coupled device (EMCCD) camera. Z stacks (0.2 μm) were collected from the entire gonad. A minimum of three germ lines was examined for each condition. Images were deconvolved using Applied Precision SoftWoRx or Nikon NIS Elements Offline batch deconvolution software employing either “Automatic3D” or “Richardson-Lucy” deconvolution modes and subsequently processed and analyzed using Fiji (ImageJ) (Wayne Rasband, NIH).

RAD-51 foci were quantified in a minimum of three germ lines of age-matched hermaphrodites (18–24 hr post-L4). As *zim-1* mutants have an extended transition zone [42], we divided germ lines into four equal zones from the beginning of the transition zone (leptotene/zygotene), as counted from the first row with three or more crescent-shaped nuclei, through diplotene (Fig 6B). The number of foci per nucleus was scored for each region.

To assess formation of RAD-51 foci following IR treatment, 18–24 hrs post-L4 worms were exposed to 10 Gy of IR; gonads were dissected 1, 4, 8, and 12 hr following IR treatment and fixed for immunofluorescence as above.

GFP::COSA-1 foci were quantified from deconvolved 3D data stacks; nuclei were scored individually through z-stacks to ensure that all foci within each individual nucleus were counted. Nuclei with features indicative of apoptosis (compact and DAPI-bright) were excluded. Foci were counted in the last five rows of pachytene nuclei as in [30].

For live cell imaging, 18–24 hr post L4 hermaphrodites were anesthetized in 1mM tetramisole and immobilized between a coverslip and an 2% agarose pad on a glass slide. Z-stacks (0.33 μm) were captured on a spinning-disk module of an inverted objective fluorescence microscope (NIH 1S10RR024543) with a 100×, NA 1.46 objective, and EMCCD camera. Z-projections of stacks were generated, cropped, and adjusted for brightness in Fiji.

Pearson’s Correlation Coefficient (PCC) was determined by drawing a Region of Interest (ROI) around a nucleus and using the co-localization function in Fiji.

### Immunoblot analysis

Whole worm lysates were generated from indicated worms; unmated *fog-2(q71)* worms were used to eliminate embryos. ~100 worms were collected, washed in M9 buffer and resuspended in equal volume of 2X Laemmli sample buffer (Bio-RAD). Lysates were resolved on 4–15% SDS-PAGE gradient gels (Bio-RAD) and transferred to Millipore Immobilon-P PVDF membranes. Membranes were blocked with 5% nonfat milk and probed with rabbit anti-GFP (1:1000; NB600-308; Novus Biologicals, Littleton, CO) and mouse anti-α-tubulin (1:1000; Sigma-Aldrich; T9026) as loading control, followed by IRDye680LT- and IRDye800-conjugated anti-rabbit and anti-mouse IgG secondary antibodies (1:20000; LI-COR Bioscience Lincoln, NE). Immunoblots were imaged on a LI-COR Odyssey Infrared Imager, signal was quantified using Fiji and normalized with the α-tubulin signal.

### RNA-mediated interference analysis

RNA-mediated interference (RNAi) was performed at 20°C, using the feeding method [113]. Cultures were plated onto NGM plates containing 25 μg/ml carbenicillin and 1 mM IPTG and were used within 2 weeks. L4 worms were transferred to RNAi plates, and resulting progeny were exposed to IR as described above. The efficiency of RNAi was tested in parallel by examining embryonic lethality.

### Meiotic mapping

Meiotic crossover frequencies and distribution were assayed utilizing single-nucleotide polymorphism (SNP) markers as in [114]. The SNP markers located at the boundaries of the chromosome domains were chosen based on data from WormBase (WS231) and [64], and are indicated in Fig 8D. The SNP markers and primers used are listed in [86]. PCR and restriction digests of single embryo lysates were performed and confirmed with additional SNPs as described in [115, 116] (Fig 8D). Statistical analyses were performed using the two-tailed Fisher's Exact test, 95% C.I., as in [117, 118]. For statistical analyses of interference we conducted χ^2^ tests on 2-by-2 contingency tables of observed and expected DCOs [119].

## Supporting information

S1 Table Genetic mapping data. (XLSX)

S2 Table Reagents used for generating CRISPR/Cas9 edited worms. (DOCX)

S3 Table Strains used in this study. (DOCX)

S1 Fig GFP::BRC-1, TagRFP-T::BRC-1 and BRD-1::GFP are functional and expressed predominantly in the germ line. A) % embryonic lethality; B) % male progeny; C) % embryonic lethality following 75 Gy IR; D) % embryonic lethality following treatment with 5mM HU for 16 hrs of indicated strains. 95% Confidence Intervals shown; * p<0.05; ** p<0.001; *** p<0.0001. *gfp*::*brc-1*, *tag-rfp-t*::*brc-1* and *brd-1*::*gfp* are not statistically different compared to WT. A minimum of 10 worms were analyzed for each condition. E) Immunoblot of whole worm extracts from WT, *gfp*::*brc-1* and *gfp*::*brc-1; fog-2* probed with rabbit anti-GFP and mouse anti-α-tubulin. Ratio determined by fluorescent intensities from three independent experiments.
(TIF)

S2 Fig In the absence of SPO-11, GFP::BRC-1 and BRD-1::GFP are enriched on a subset of chromosomes. A) Number of GFP::BRC-1 foci in indicated mutants in Proliferative Zone, Transition Zone and Early Pachytene. Number of foci examined in a minimum of 3 germ lines: PZ: WT (n = 412); *spo-11* (n = 177); *rad-51*(n = 114); *msh-5* (n = 175); *syp-1* (n = 140); *zim-1* (n = 142); TZ: WT (n = 287); *spo-11* (n = 103); *rad-51*(n = 52); *msh-5* (n = 94); *syp-1* (n = 83); *zim-1* (n = 112); EP: WT (n = 202); *spo-11* (n = 106); *rad-51*(n = 57); *msh-5* (n = 57); *syp-1* and *zim-1* had too many foci to accurately count. *** p<0.0001. B) Late pachytene region of the germ line stained with anti-GFP (green) and anti-phosphoSYP-4 (SYP-4P) (red) and counterstained with DAPI. Scale bar = 10 μm. C) High-magnification images of live *C*. *elegans* expressing BRD-1::GFP in the *spo-11* background. Images are projections through half of the gonad. PZ = Proliferative Zone, TZ = Transition Zone, EP = Early Pachytene, MP = Mid Pachytene, LP = Late Pachytene, DP = Diplotene, DK = Diakinesis. Scale bar = 5 μm.
(TIF)

S3 Fig *brc-1* and *brd-1* mutant alleles and meiotic progression. A) Genomic regions of *brc-1* and *brd-1* from WormBase Version: WS265 (https://wormbase.org/#012-34-5), with the region deleted in the different alleles indicated. Color dotted lines indicate the resulting splicing of *brd-1*(*ok1623)* (pink; splicing of exon 7–12, which introduces a stop codon and results in a 343 a. a. protein) and *brd-1(dw1)* (orange; cryptic splice site within intron 11 spliced to exon 12, resulting in a 375 a. a. protein) as determined by cDNA analysis. B) High-magnification images of live *brc-1(tm1145)* worms expressing BRD-1::GFP (PZ = Proliferative Zone, TZ = Transition Zone, EP = Early Pachytene, MP = Mid Pachytene, LP = Late Pachytene, DP = Diplotene, DK = Diakinesis). Scale bar = 5 μm. C) Indicated germ lines stained with antibodies against SUN-1 S12P (green) and counterstained with DAPI (blue). Numbers beneath genotype indicate the percentage of cell rows with SUN-1 S12P staining normalized to gonad length as in [49]; 3 germ lines were examined. Images are projections through half of the gonad. Scale bar = 20 μm.
(TIF)

S4 Fig Inactivation of *brc-1* or *brd-1* alters pattern of RAD-51 foci in mid-late pachytene in chromosome synapsis mutants. A) Box whisker plots show average number of RAD-51 foci per nucleus in the different zones of meiotic prophase (see Fig 6B). Horizontal line of each box indicates the median, the top and bottom of the box indicates medians of upper and lower quartiles, lines extending above and below boxes indicate standard deviation and individual data points are outliers from 5–95%. Numbers of nuclei scored in each zone for WT: 1 = 186; 2 = 343; 3 = 292; 4 = 166; *brc-1(tm1145) brd-1(dw1)*: 1 = 233; 2 = 303; 3 = 261; 4 = 68; *brd-1(ok1623)*: 1 = 186; 2 = 135; 3 = 162; 4 = 117. * p<0.05; ** p<0.001; *** p<0.0001. B) Dissected germ lines from *brd-1(ok1623); zim-1*, *brc-1(tm1145) brd-1(dw1); syp-1*, *syp-1*, *brd-1(dw1); zim-1*, *brc-1(tm1145) brd-1(dw1)*; *zim-1*, and *brc-1(xoe4); zim-1* worms stained with anti-RAD-51 (red) and counterstained with DAPI (blue); white bracket indicates region of reduced RAD-51 foci. A minimum of 4 germ lines were imaged for each genotype. Full projections of the gonads are shown. Scale bar = 20 μm. C) Mid-late pachytene region of gonad from *gfp*::*rpa-1* and *brc-1(tm1145) brd-1(dw1); gfp*::*rpa-1* worms stained with anti-RAD-51 (red) and imaged for GFP::RPA-1 fluorescence (green), counterstained with DAPI (blue). Images are projections through half of the gonad. Scale bar = 8 μm.
(TIF)

S5 Fig COSA-1 foci in synapsis mutants in the presence and absence of BRC-1. Late pachytene region of the germ line in indicated mutants expressing GFP::COSA-1 (green) and counterstained with DAPI (blue). Images are projections through half of the gonad. Scale bar = 5 μm.
(TIF)
