# Supplementary material for: The tumor suppressor BRCA1-BARD1 complex localizes to the synaptonemal complex and regulates recombination under meiotic dysfunction in Caenorhabditis elegans
Source: PLoS Genet. 2018 Nov 1;14(11):e1007701. doi: 10.1371/journal.pgen.1007701 (PMC6211623; doi:10.1371/journal.pgen.1007701)
Supplement: S2 Table — (DOCX) [file pgen.1007701.s002.docx]

S2 Table: Reagents used for generating CRISPR/Cas9 edited worms

| genotype | sgRNA | repair template | forward primer for genotyping | reverse primer for genotyping |
| --- | --- | --- | --- | --- |
| *gfp::brc-1* | AGATGGCAGATGTTGCACTG | homology arms inserted into plasmid pDD282. * | TTTGTTTGAACGGAACCTTGC | AATAGCCCAAAAAGGCCGAA |
|  |  | 5' homology arm,  forward primer: ACGTTGTAAAACGACGGCCAGTCGCCGGCATTTAGCAGCTAAACTAACTGAAAACTAC |  |  |
|  |  | reverse primer: CTGAAATTAAATTTTAAACAAATAACATTTC |  |  |
|  |  | 3' homology arm,  forward primer: CGTGATTACAAGGATGACGATGACAAGAGAGGAGCATCGGGAGCCTCAGGAGCATCGATGGCAGATGTTGCACTGAGAATCACA |  |  |
|  |  | reverse primer:  TCACACAGGAAACAGCTATGACCATGTTATGCAGTCCGCTATGATTTTCAGTCAGAAATTAGC |  |  |
| *TagRFP-T::brc-1* | AGATGGCAGATGTTGCACTG | same homology arms as in gfp::brc-1 inserted into plasmid pDD284. * | TTTGTTTGAACGGAACCTTGC | AATAGCCCAAAAAGGCCGAA |
| *brd-1::gfp* | TGGTTAATAGAGGCGATTCT | homogy arms inserted into plasmid pDD282. * | CGCAAGGGTGCAGTATGAGA | AAGAAGGACCACCTTGCACC |
|  |  | 5' homology arm,  forward primer: ACGTTGTAAAACGACGGCCAGTCGCCGGCAGTCGAGATTGGCTGAGAGCG |  |  |
|  |  | reverse primer: CATCGATGCTCCTGAGGCTCCCGATGCTCCATGTGGTGGTGTTGTGATGGAG |  |  |
|  |  | 3' homology arm,  forward primer: CGTGATTACAAGGATGACGATGACAAGAGATAACCATTTTCCGTTTTAAAATATT |  |  |
|  |  | reverse primer: GGAAACAGCTATGACCATGTTATCGATTTCATGGTACATACTCCTTCACTACTGAC |  |  |
| *brc-1(xoe4)* | 1: AGATGGCAGATGTTGCACTG | TTGATTTTTCAATTAAAATGAAATGTTATTTGTTTAAAATTTAATTTCAGACAAAGCTGTCGATTCGATAGGCTGCCTGCAGGATTAATTTTCCATTTCT | TTTGTTTGAACGGAACCTTGC | GAGCTAGCGCAAAATCAACG |
|  | 2: GACAGCTTTGTAAGGTTGAG |  |  |  |
|  | 3: ACAGCTTTGTAAGGTTGAGT |  |  |  |

* additional silent mutations introduced to change PAM or sgRNA sequence to avoid cutting by Cas9.
