# Supplementary material for: The tumor suppressor BRCA1-BARD1 complex localizes to the synaptonemal complex and regulates recombination under meiotic dysfunction in Caenorhabditis elegans
Source: PLoS Genet. 2018 Nov 1;14(11):e1007701. doi: 10.1371/journal.pgen.1007701 (PMC6211623; doi:10.1371/journal.pgen.1007701)
Supplement: S3 Table — (DOCX) [file pgen.1007701.s003.docx]

S3 Table. Strains used in this study

| Name | Genotype | Source |
| --- | --- | --- |
| JEL515 | *gfp::brc-1 III* | This study |
| JEL657 | *brd-1::gfp III* | This study |
| JEL587 | *gfp::brc-1 III; fog-2(q71) V* | This study |
| JEL539 | *tag-rfp-t::brc-1 III* | This study |
| JEL542 | *meIs8[unc-119(+) pie-1promoter::gfp::cosa-1] II; tag-rfp-t::brc-1 III* | This study |
| JEL520 | *gfp::brc-1 brd-1(ok1623) III* | This study |
| JEL678 | *brd-1::gfp brc-1(tm1145) III* | This study |
| JEL694 | *gfp::brc-1 III; him-8 mCherry::his58 IV* | This study |
| JEL543 | *gfp::brc-1; spo-11(ok79)/nT1[unc-?(n754) let-?] (IV;V)* | This study |
| JEL611 | *gfp::brc-1; rad-51(ok2218)/nT1 [qls] (IV;V)* | This study |
| JEL685 | *gfp::brc-1; msh-5(me23)/nT1[unc-?(n754) let-?] (IV;V)* | This study |
| JEL527 | *gfp::brc-1; syp-1(me17)/nT1[unc-?(n754) let-? qls50] (IV;V)* | This study |
| JEL519 | *gfp::brc-1; zim-1(tm1813)* | This study |
| JEL684 | *brd-1::gfp; spo-11(ok79)/nT1[unc-?(n754) let-?] (IV;V)* | This study |
| DW102 | *brc-1(tm1145) brd-1(dw1) III* | Shohei Mitani |
| RB1426 | *brd-1(ok1623) III* | OMRF knockout  group |
| JEL729 | *brc-1(tm1145) III* | This study |
| JEL730 | *brc-1(xoe4) III* | This study |
| DW103 | *brd-1(dw1) III* | Shohei Mitani |
| JEL187 | *brc-1(tm1145) III; zim-1(tm1813) IV* | This study |
| JEL504 | *brd-1(ok1623); zim-1(tm1813)* | This study |
| JEL273 | *gfp::rpa-1 II* | Sonneville et al.,  2012 |
| JEL734 | *brd-1(dw1) III; zim-1(tm1813) IV* | This study |
| JEL749 | *brc-1(xoe4) III; zim-1(tm1813) IV* | This study |
| JEL750 | *brc-1(tm1145) brd-1(dw1) III; zim-1(tm1813) IV* | This study |
| JEL565 | *brc-1(tm1145) brd-1(dw1) III; gfp::rpa-1* | This study |

| JEL499 | *gfp::rpa-1 II; zim-1(tm1813) IV;* | This study |
| --- | --- | --- |
| JEL498 | *gfp::rpa-1 II; brc-1(tm1145) III; zim-1(tm1813) IV;* | This study |
| AV106 | *spo-11(ok79)/nT1[unc-?(n754) let-?] (IV;V)* | Dernburg et al., 1998 |
| JEL582 | *brc-1(tm1145) brd-1(dw1) III; spo-11(ok79)/nT1[unc-?(n754) let-?]*  *(IV;V)* | This study |
| AV630 | *meIs8[unc-119(+) pie-1promoter::gfp::cosa-1] II* | Yokoo et al., 2012 |
| JEL301 | *meIs8[unc-119(+) pie-1promoter::gfp::cosa-1] II; brc- 1(tm1145) brd-1(dw1) III* | This study |
| JEL698 | *meIs8[unc-119(+) pie-1promoter::gfp::cosa-1] II; brd- 1(1623) III* | This study |
| JEL747 | *meIs8[unc-119(+) pie-1promoter::gfp::cosa-1] II; brc- 1(tm1145) III* | This study |
| JEL477 | *meIs8[unc-119(+) pie-1promoter::gfp::cosa-1] II; zim- 1(tm1813)* | This study |
| JEL490 | *meIs8[unc-119(+) pie-1promoter::gfp::cosa-1] II; brc- 1(tm1145) III; zim-1(tm1813) IV* | This study |
| JEL691 | *meIs8[unc-119(+) pie-1promoter::gfp::cosa-1] II; brd- 1(ok1623) III; zim-1(tm1813) IV* | This study |
| JEL480 | *meIs8[unc-119(+) pie-1promoter::gfp::cosa-1] II; syp- 1(me17)/nT1[unc-?(n754) let-? qls50] (IV;V)* | This study |
| JEL485 | *meIs8[unc-119(+) pie-1promoter::gfp::cosa-1] II; brc- 1(tm1145) brd-1(dw1) III; syp-1(me17)/nT1[unc-?(n754) let-? qls50] (IV;V)* | This study |
| JEL748 | *meIs8[unc-119(+) pie-1promoter::gfp::cosa-1] II; brc- 1(tm1145) brd-1(dw1) III; zim-1(tm1813) IV* | This study |
