# Supplementary figures and images for: The tumor suppressor BRCA1-BARD1 complex localizes to the synaptonemal complex and regulates recombination under meiotic dysfunction in Caenorhabditis elegans
Source: PLoS Genet. 2018 Nov 1;14(11):e1007701. doi: 10.1371/journal.pgen.1007701 (PMC6211623; doi:10.1371/journal.pgen.1007701)

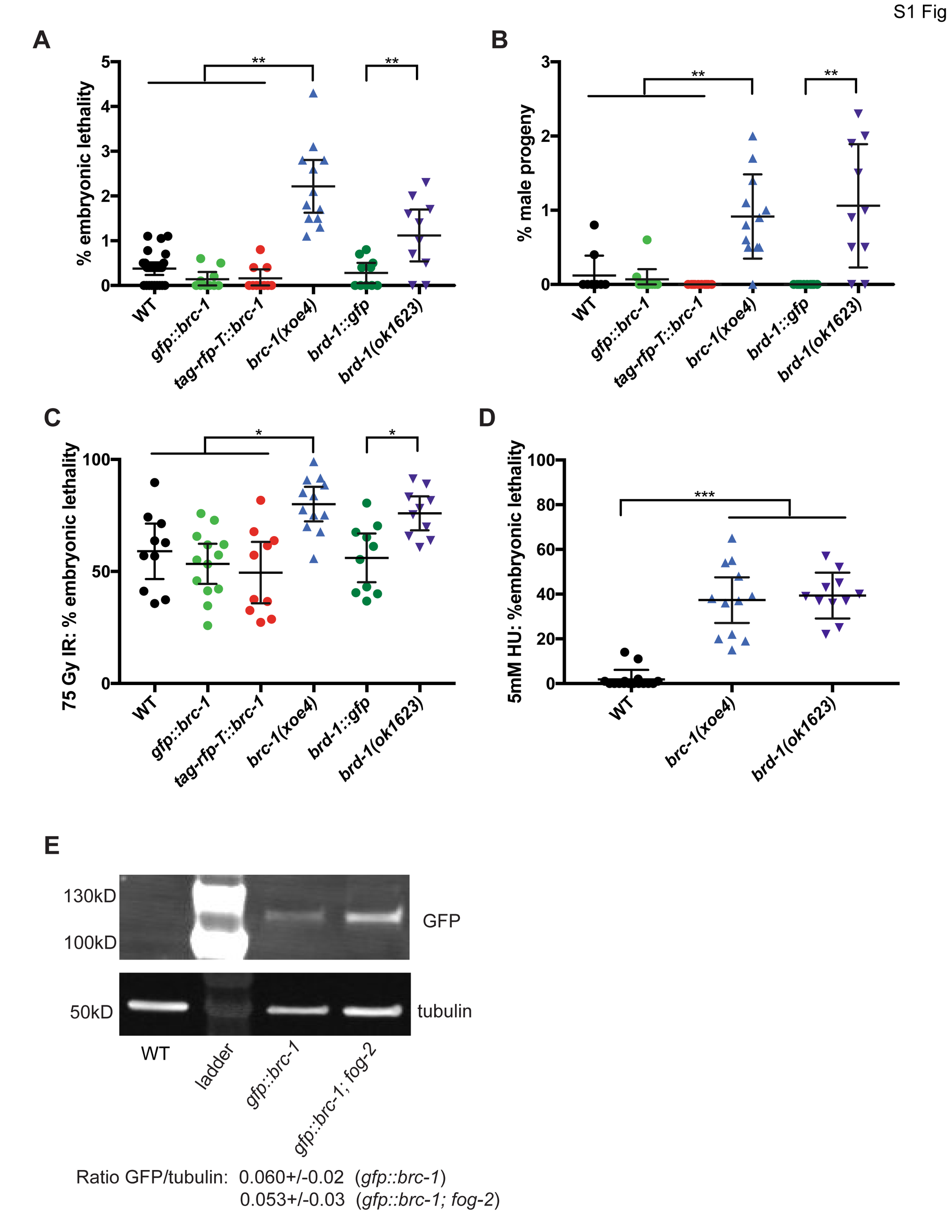

Supplement: S1 Fig — A) % embryonic lethality; B) % male progeny; C) % embryonic lethality following 75 Gy IR; D) % embryonic lethality following treatment with 5mM HU for 16 hrs of indicated strains. 95% Confidence Intervals shown; * p<0.05; ** p<0.001; *** p<0.0001. gfp::brc-1, tag-rfp-t::brc-1 and brd-1::gfp are not statistically different compared to WT. A minimum of 10 worms were analyzed for each condition. E) Immunoblot of whole worm extracts from WT, gfp::brc-1 and gfp::brc-1; fog-2 probed with rabbit anti-GFP and mouse anti-α-tubulin. Ratio determined by fluorescent intensities from three independent experiments. (TIF) [file pgen.1007701.s004.tif]

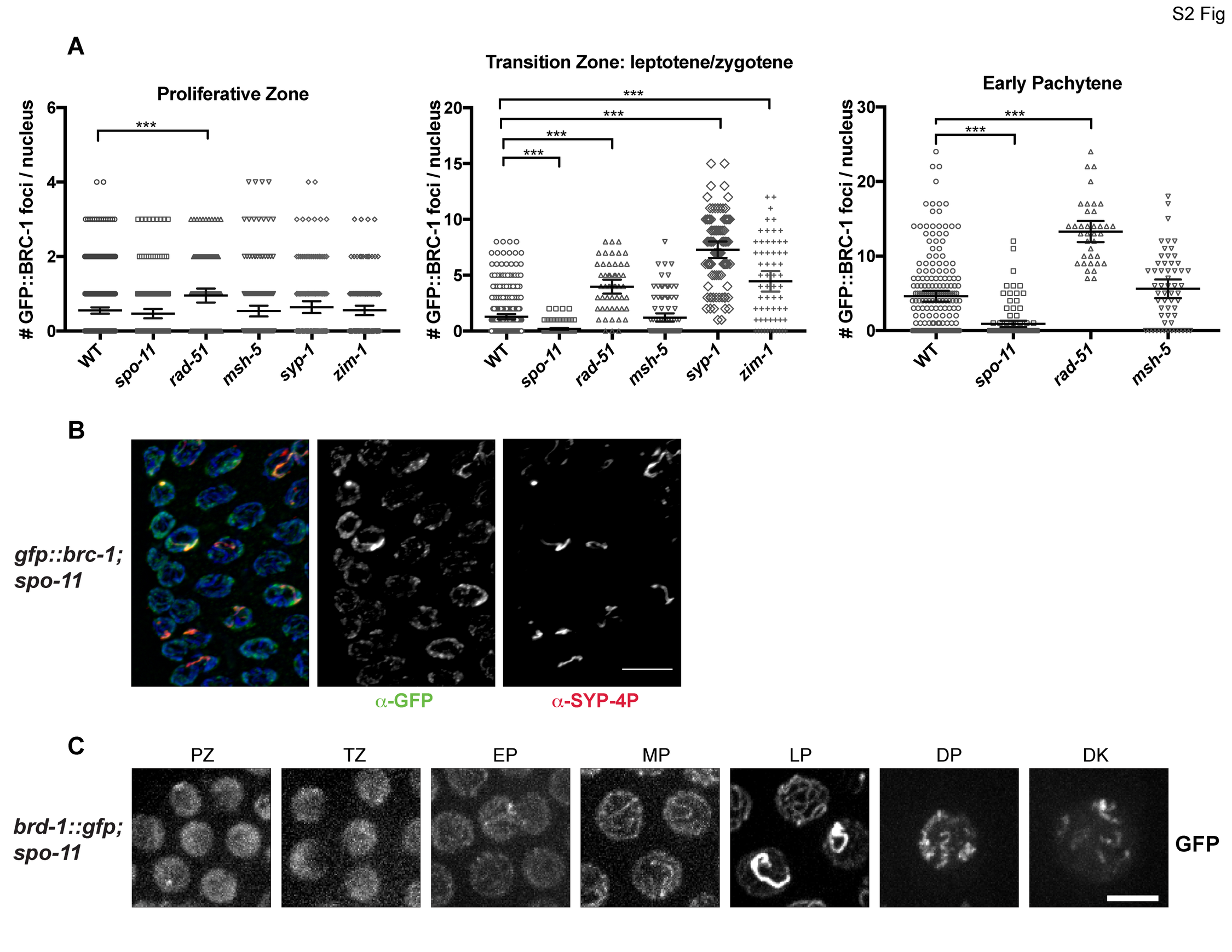

Supplement: S2 Fig — A) Number of GFP::BRC-1 foci in indicated mutants in Proliferative Zone, Transition Zone and Early Pachytene. Number of foci examined in a minimum of 3 germ lines: PZ: WT (n = 412); spo-11 (n = 177); rad-51(n = 114); msh-5 (n = 175); syp-1 (n = 140); zim-1 (n = 142); TZ: WT (n = 287); spo-11 (n = 103); rad-51(n = 52); msh-5 (n = 94); syp-1 (n = 83); zim-1 (n = 112); EP: WT (n = 202); spo-11 (n = 106); rad-51(n = 57); msh-5 (n = 57); syp-1 and zim-1 had too many foci to accurately count. *** p<0.0001. B) Late pachytene region of the germ line stained with anti-GFP (green) and anti-phosphoSYP-4 (SYP-4P) (red) and counterstained with DAPI. Scale bar = 10 μm. C) High-magnification images of live C. elegans expressing BRD-1::GFP in the spo-11 background. Images are projections through half of the gonad. PZ = Proliferative Zone, TZ = Transition Zone, EP = Early Pachytene, MP = Mid Pachytene, LP = Late Pachytene, DP = Diplotene, DK = Diakinesis. Scale bar = 5 μm. (TIF) [file pgen.1007701.s005.tif]

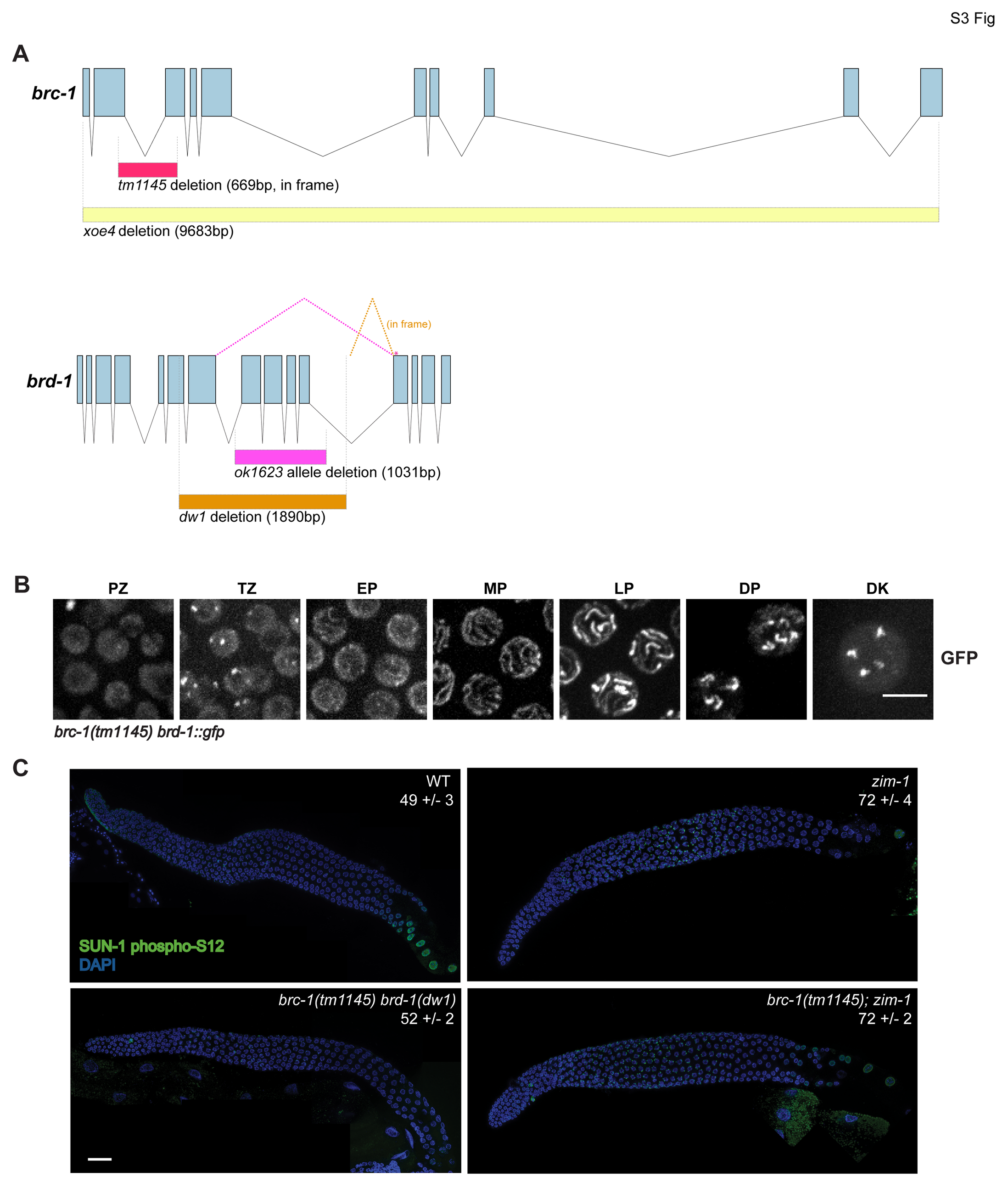

Supplement: S3 Fig — A) Genomic regions of brc-1 and brd-1 from WormBase Version: WS265 (https://wormbase.org/#012-34-5), with the region deleted in the different alleles indicated. Color dotted lines indicate the resulting splicing of brd-1(ok1623) (pink; splicing of exon 7–12, which introduces a stop codon and results in a 343 a. a. protein) and brd-1(dw1) (orange; cryptic splice site within intron 11 spliced to exon 12, resulting in a 375 a. a. protein) as determined by cDNA analysis. B) High-magnification images of live brc-1(tm1145) worms expressing BRD-1::GFP (PZ = Proliferative Zone, TZ = Transition Zone, EP = Early Pachytene, MP = Mid Pachytene, LP = Late Pachytene, DP = Diplotene, DK = Diakinesis). Scale bar = 5 μm. C) Indicated germ lines stained with antibodies against SUN-1 S12P (green) and counterstained with DAPI (blue). Numbers beneath genotype indicate the percentage of cell rows with SUN-1 S12P staining normalized to gonad length as in [49]; 3 germ lines were examined. Images are projections through half of the gonad. Scale bar = 20 μm. (TIF) [file pgen.1007701.s006.tif]

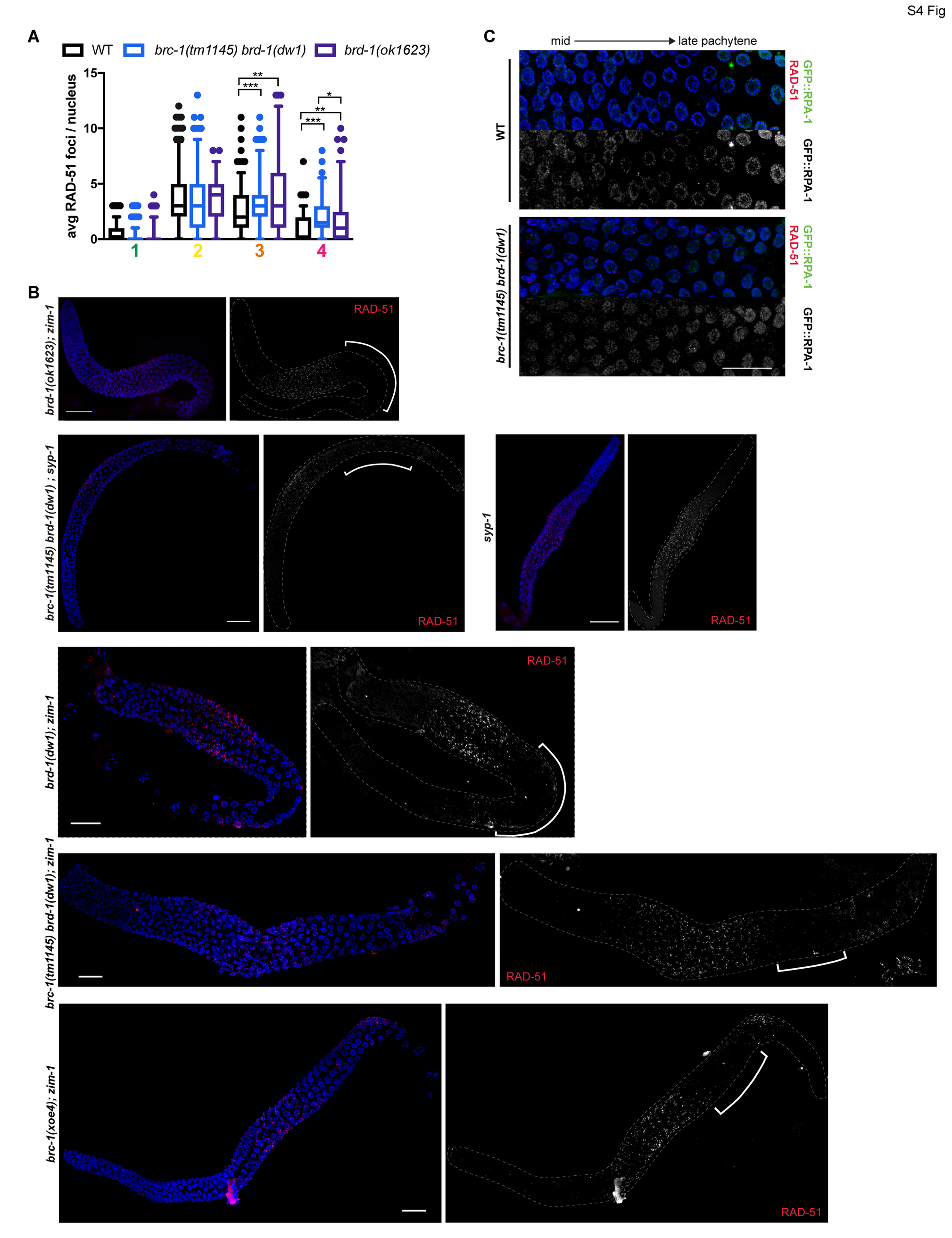

Supplement: S4 Fig — A) Box whisker plots show average number of RAD-51 foci per nucleus in the different zones of meiotic prophase (see Fig 6B). Horizontal line of each box indicates the median, the top and bottom of the box indicates medians of upper and lower quartiles, lines extending above and below boxes indicate standard deviation and individual data points are outliers from 5–95%. Numbers of nuclei scored in each zone for WT: 1 = 186; 2 = 343; 3 = 292; 4 = 166; brc-1(tm1145) brd-1(dw1): 1 = 233; 2 = 303; 3 = 261; 4 = 68; brd-1(ok1623): 1 = 186; 2 = 135; 3 = 162; 4 = 117. * p<0.05; ** p<0.001; *** p<0.0001. B) Dissected germ lines from brd-1(ok1623); zim-1, brc-1(tm1145) brd-1(dw1); syp-1, syp-1, brd-1(dw1); zim-1, brc-1(tm1145) brd-1(dw1); zim-1, and brc-1(xoe4); zim-1 worms stained with anti-RAD-51 (red) and counterstained with DAPI (blue); white bracket indicates region of reduced RAD-51 foci. A minimum of 4 germ lines were imaged for each genotype. Full projections of the gonads are shown. Scale bar = 20 μm. C) Mid-late pachytene region of gonad from gfp::rpa-1 and brc-1(tm1145) brd-1(dw1); gfp::rpa-1 worms stained with anti-RAD-51 (red) and imaged for GFP::RPA-1 fluorescence (green), counterstained with DAPI (blue). Images are projections through half of the gonad. Scale bar = 8 μm. (TIF) [file pgen.1007701.s007.tif]

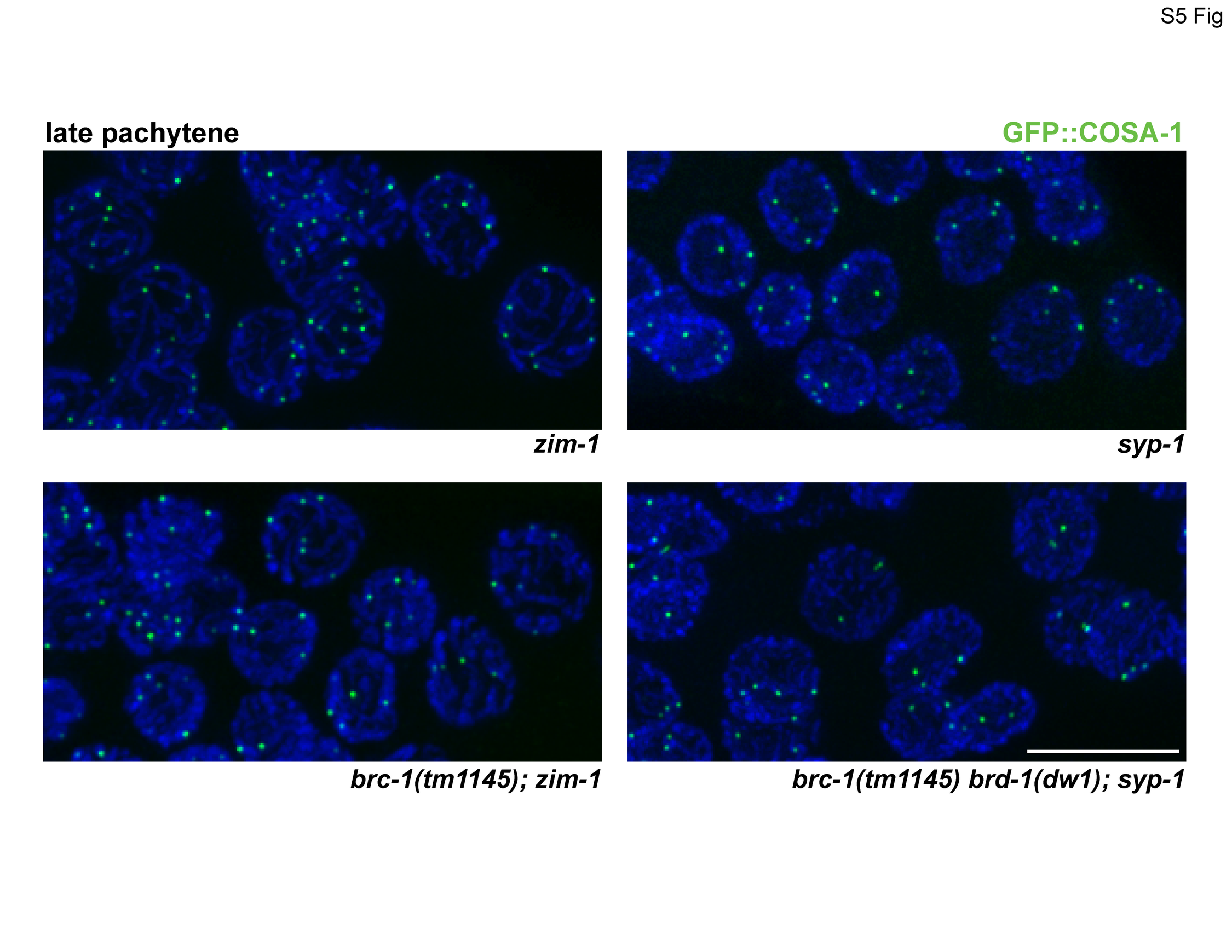

Supplement: S5 Fig — Late pachytene region of the germ line in indicated mutants expressing GFP::COSA-1 (green) and counterstained with DAPI (blue). Images are projections through half of the gonad. Scale bar = 5 μm. (TIF) [file pgen.1007701.s008.tif]
